# Full-length ribosome density prediction by a multi-input and multi-output model

**DOI:** 10.1371/journal.pcbi.1008842

**Published:** 2021-03-26

**Authors:** Tingzhong Tian, Shuya Li, Peng Lang, Dan Zhao, Jianyang Zeng

**Affiliations:** 1 Institute for Interdisciplinary Information Sciences, Tsinghua University, Beijing, China; 2 MOE Key Laboratory of Bioinformatics, Tsinghua University, Beijing, China; University of Wisconsin, Madison, UNITED STATES

## Abstract

Translation elongation is regulated by a series of complicated mechanisms in both prokaryotes and eukaryotes. Although recent advance in ribosome profiling techniques has enabled one to capture the genome-wide ribosome footprints along transcripts at codon resolution, the regulatory codes of elongation dynamics are still not fully understood. Most of the existing computational approaches for modeling translation elongation from ribosome profiling data mainly focus on local contextual patterns, while ignoring the continuity of the elongation process and relations between ribosome densities of remote codons. Modeling the translation elongation process in full-length coding sequence (CDS) level has not been studied to the best of our knowledge. In this paper, we developed a deep learning based approach with a multi-input and multi-output framework, named RiboMIMO, for modeling the ribosome density distributions of full-length mRNA CDS regions. Through considering the underlying correlations in translation efficiency among neighboring and remote codons and extracting hidden features from the input full-length coding sequence, RiboMIMO can greatly outperform the state-of-the-art baseline approaches and accurately predict the ribosome density distributions along the whole mRNA CDS regions. In addition, RiboMIMO explores the contributions of individual input codons to the predictions of output ribosome densities, which thus can help reveal important biological factors influencing the translation elongation process. The analyses, based on our interpretable metric named codon impact score, not only identified several patterns consistent with the previously-published literatures, but also for the first time (to the best of our knowledge) revealed that the codons located at a long distance from the ribosomal A site may also have an association on the translation elongation rate. This finding of long-range impact on translation elongation velocity may shed new light on the regulatory mechanisms of protein synthesis. Overall, these results indicated that RiboMIMO can provide a useful tool for studying the regulation of translation elongation in the range of full-length CDS.

## Author summary

Translation elongation is a process in which amino acids are linked into proteins by ribosomes in cells. Translation elongation rates along the mRNAs are not constant, and are regulated by a series of mechanisms, such as codon rarity and mRNA stability. In this study, we modeled the translation elongation process at a full-length coding sequence level and developed a deep learning based approach to predict the translation elongation rates from mRNA sequences, through extracting the regulatory codes of elongation rates from the contextual sequences. The analyses, based on our interpretable metric named codon impact score, for the first time (to the best of our knowledge), revealed that in addition to the neighboring codons of the ribosomal A sites, the remote codons may also have an important impact on the translation elongation rates. This new finding may stimulate additional experiments and shed light on the regulatory mechanisms of protein synthesis.

## Introduction

Translation elongation plays an essential role in protein synthesis, in which the nucleotide triplets on message RNAs (mRNAs) are deciphered by ribosomes into peptides [1]. During elongation, transfer RNAs (tRNAs) carrying the corresponding amino acids are recruited to the ribosomal A site and the amino acid with correct codon-anticodon complementarity is appended to the end of nascent peptide until the whole polypeptide is produced. Single amino acid can be encoded by multiple synonymous codons, and the elongation rates on these synonymous codons are generally not evenly distributed [2]. Rare codons are more likely to reduce the elongation rates, sometimes even causing pausing (also termed ribosome stalling), which is generally associated with mRNA degradation, low protein expression or protein misfolding [3–5].

Ribosome profiling techniques have provided great opportunities for revealing the translation elongation dynamics by sequencing the reads of ribosome protected fragments (RPFs) captured during elongation at codon resolution [6, 7]. The normalized RPF counts can directly reflect the ribosome density distribution along the coding sequence (CDS), where higher densities generally correspond to slow elongation rates and vise versa [8]. In order to capture the contextual determinants of elongation rates, several computational methods have been proposed to model the translation elongation dynamics from mRNA sequences. O’Connor et al. introduced a ribosome density normalization method, named RUST, to measure the positional effects of individual codons on the elongation rates at the A site [9]. Liu et al. used a sparse regression model with kernel smoothing, named riboShape, to capture the codon-level contributions to the translation speeds at multiple scales of wavelet decomposition components [10]. Zhang et al. proposed a deep learning based method, named ROSE, to predict the ribosome stalling sites from mRNA sequences [11]. Tunney et al. developed another deep learning based model, named iXnos, to reconstruct the ribosome density distributions from local sequence contexts near the A site codons [12].

Despite the above efforts into modeling the ribosome density distributions, most of them only focus on the local contexts of A site codons and ignore the potential influence of the whole CDS. As the elongation process is continuous, it is natural to model the translation elongation at full-length CDS level. In addition, it remains largely unclear whether remote codons can also affect the ribosome elongation rate of the codon at the ribosomal A site. Thus, modeling the translation elongation process at full-length CDS level can capture the (possibly existing) long-range associations between remote codons resided in the transcript. In this paper, we proposed a deep learning based model with a multi-input and multi-output strategy, named RiboMIMO, to model the translation elongation rates of full-length transcripts. RiboMIMO adopts a sequence-to-sequence manner for the first time (to the best of our knowledge) to model and predict the ribosome density distributions, and greatly outperforms the state-of-the-art baseline methods.

RiboMIMO takes the full-length CDS as input, which can be of arbitrary length ranging from a few hundred to several thousand codons. This multi-input design ensures our model to capture any possible influence on the elongation rate from any codon position. Also, RiboMIMO predicts the ribosome density distributions of all codons within a CDS sequence simultaneously. This multi-output setting guarantees that the relations between ribosome densities of adjacent codons can be effectively captured from the ribosome profiling data. Therefore, RiboMIMO has the ability to capture long-range determinants of ribosome densities. Furthermore, RiboMIMO optimizes dual-task losses to improve the prediction performance. In addition to a basic regression task, RiboMIMO introduces an additional classification task for predicting the discretized ribosome density distribution of individual codons, which can also help improve the ribosome density prediction task. We also define a new metric, named the codon impact score (CIS) based on the RiboMIMO framework, for measuring the influence of a codon to the predicted ribosome density of another codon at any position. The impact map of CIS shows strong consistence with the existing studies in the literature, and the new findings of long-range influence of ribosome densities may shed light on the regulatory mechanisms of translation elongation.

## Materials and methods

### Data preprocessing and problem formulation

We trained and evaluated our model on four ribosome profiling datasets collected from *Escherichia coli* (one from GEO: GSE72899, denoted as Mohammad16, two from GEO: GSE119104, denoted as Mohammad19-1 and Mohammad19-2, respectively) [13, 14] and *Saccharomyces cerevisiae* (GEO: GSE53268, denoted as Subtelny14)) [15]. For the Subtelny14 dataset, we first downloaded the raw ribosome profiling data (GSM1289257) from the GEO database and then aligned the reads to yeast transcriptome following the same protocol as described in [15]. Specifically, the raw reads obtained from the GEO database were trimmed to remove the adapter sequences. Then the reads mapped to the ribosomal RNAs (rRNAs) or non-coding RNAs (ncRNAs) of the yeast genome were removed using bowtie2 [16]. The remaining clean reads were then aligned to the yeast transcriptome using hisat2 [17]. The ribosome densities of A sites were then obtained from the aligned data. For the Mohammad16 dataset, the aligned ribosome density data were directly downloaded from the GWIPS-viz [18] database and then averaged over three replicates. For the Mohammad19 datasets, the raw reads (GSM3358140 and GSM3358142) were downloaded from the GEO database, and the ribosome densities were obtained using the source code provided by the original authors. The source code and all the processed data can be found in our GitHub repository (https://github.com/tiantz17/RiboMIMO).

In this paper, we use light symbols (e.g., *x*) to denote scalar variables, bold symbols (e.g., ***x***) to denote vector or tensor variables and curlicue symbols (e.g., C) to denote sets. Let $x_{g}=\left(x_{g,1},\cdots ,x_{g,n_{g}}\right)$ denote an mRNA sequence of gene *g* with a length of *n*_*g*_ codons, where $x_{g,i}\in C$ stands for one of 64 codon types, and C stands for the set of all codon types. The average count of ribosome footprint reads over biological replicates for the codon at position *i* of gene *g* from the ribosome profiling data is represented as $r_{g,i}\in R$. Then the sequencing coverage of gene *g* is defined as $c_{g}=\sum_{i=1}^{n_{g}}I\left(r_{g,i}>0.5\right)/n_{g}$, where I(·) stands for the indicator function. The averaged ribosome density of gene *g*, denoted by ${\bar{r}}_{g}$, is defined as the average number of ribosome footprint reads in the transcript, that is,

$$\begin{array}{c} {\bar{r}}_{g}=\frac{\sum_{i=1}^{n_{g}}r_{g,i}\cdot I\left(r_{g,i}>0.5\right)}{\sum_{i=1}^{n_{g}}I\left(r_{g,i}>0.5\right)}. \end{array}$$ (1)

Note that here we used only positions with non-zero read counts in gene *g* to calculate *c*_*g*_ and ${\bar{r}}_{g}$ mainly for the purpose of reducing the potential bias introduced by zero reads, which are regarded as unobserved data rather than zero densities. Here, we used a threshold of 0.5 to filter out zero counts, which was also adopted in [19].

In order to maintain the high quality of the samples and remove the poorly sequenced transcripts, here we followed the same strategy as in [20] and only kept those genes with sequencing coverage *c*_*g*_ higher than 60%. Note that one gene with high sequencing coverage generally corresponds to a high averaged ribosome density (S1 Fig). Thus, using sequence coverage alone as a filtering criterion is sufficient for selecting the high-quality data. The statistics of the datasets used in this paper can be found in S1 Table.

**Fig 1:**
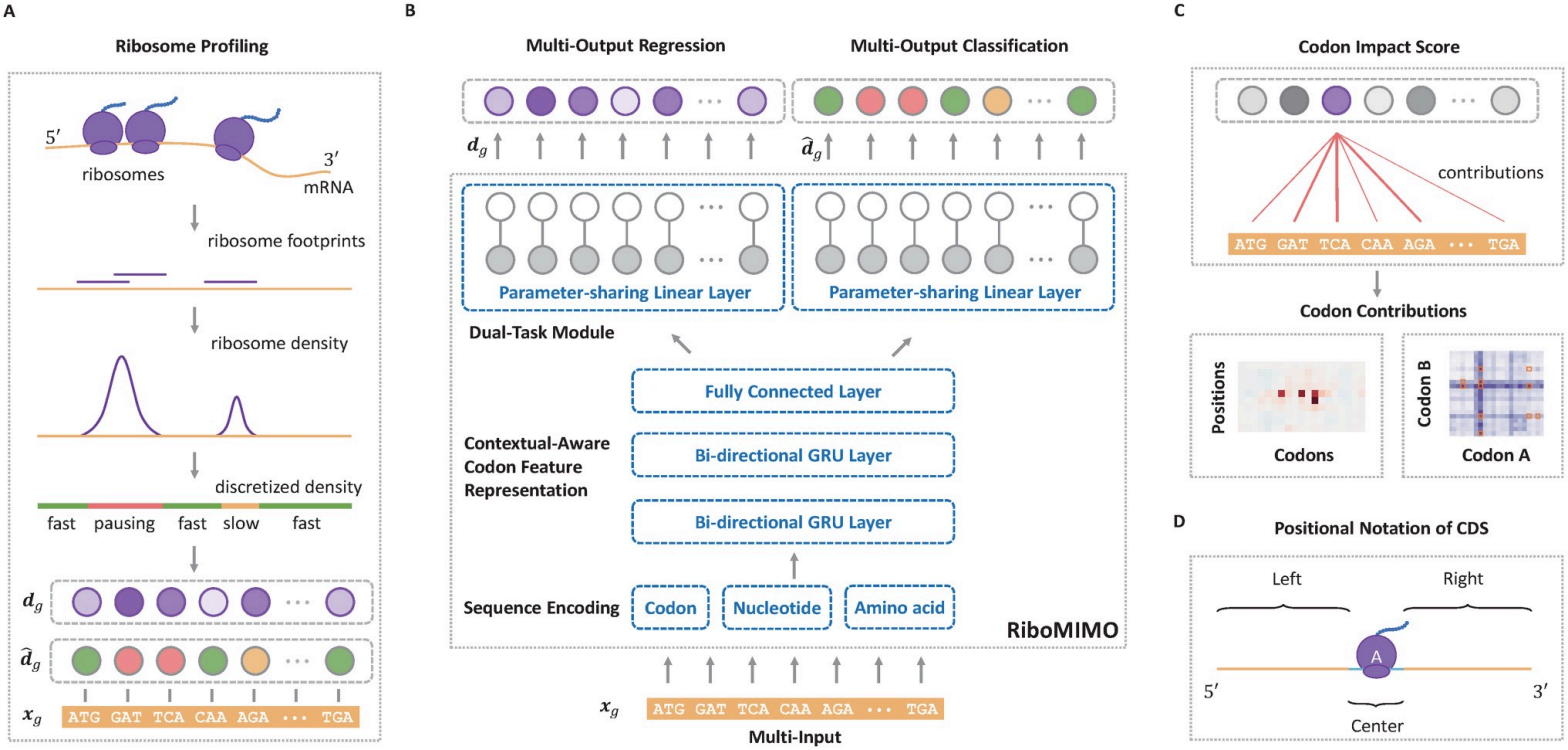
Overview of the RiboMIMO pipeline. (A). Schematic illustration of the input ribosome profiling data. The ribosome protected fragments were first sequenced and then aligned to the genome. Next, the ribosome densities are obtained after eliminating the bias of mRNA abundance levels. After that, the ribosome densities are categorized into three classes, including “fast”, “slow” and “pausing”. Both the ribosome densities ***d***_*g*_ and the corresponding discretized labels ${\hat{d}}_{g}$ are used as the output targets of our model while the CDS sequence ***x***_*g*_ is used as the input. (B). The RiboMIMO architecture, which includes encoding, feature embedding and dual-task modules. The feature embedding module employs a deep neural network to learn a context-aware feature representation for each codon based on a message propagation mechanism along the input full-length CDS sequence through a bi-directional gated recurrent unit (Bi-GRU) network, followed by a fully connected network. The dual-task module consists of two sub-modules, one is a basic regression task by a parameter-sharing single-layer neural network, while the other is a classification task by another parameter-sharing single-layer neural network. (C). The codon impact score (CIS) introduced by our RiboMIMO framework for measuring the contribution of a codon feature to the predicted ribosome density of the A site of another codon position within the CDS, or specific impact of any codon pair to the elongation rate of the transcript. (D). The positional notation for regions of a CDS sequence relative to the ribosomal A site. The region of ribosome footprints near the A site is denoted as “center”, with a length of 10 codons ranging from −5 to + 4 positions, where the index of the A site is 0. The rest of the CDS sequence is separated into “left” and “right” regions, which range from the start codon to −6 position at 5′ end, and + 5 position to the stop codon at 3′ end, respectively.

Next, we normalized the counts of ribosome footprint reads by the averaged ribosome density of the same gene, and obtained the ribosome density distribution $d_{g}=\left(d_{g,1},\ldots ,d_{g,n_{g}}\right)$ of gene *g* (shown in Fig 1A), that is,

$$\begin{array}{c} d_{g,i}=r_{g,i}/{\bar{r}}_{g},\;i=1,\cdots ,n_{g}. \end{array}$$ (2)

Our dataset $D=\{\left(x_{g},d_{g}\right),g\in G\}$ consists of the mRNA sequence $x_{g}\in C^{n_{g}}$ of each gene *g* as well as its corresponding ribosome density distribution $d_{g}\in R^{n_{g}}$, where G stands for the set of genes. Here, ***x***_*g*_ starts with the start codon (i.e., *x*_*g*,1_ = ATG) and ends with one of the three stop codons (i.e., $x_{g,n_{g}}\in \left\{\text{TAA},\text{TAG},\text{TGA}\right\}$). Our goal is to predict the ribosome density distribution ***d***_*g*_ given an input mRNA sequence ***x***_*g*_.

### The RiboMIMO framework

The name “MIMO” in our RiboMIMO approach refers to the **m**ulti-**i**nput and **m**ulti-**o**utput framework for modeling ribosome density distributions, which is a specially designed characteristic for our task. The RiboMIMO architecture is shown in Fig 1B, which can be divided into three modules. First, the input mRNA sequence of a gene is fed into the encoding module and converted into machine readable codes. Then, in the feature embedding module, a deep neural network learns a context-aware feature representation for each codon based on the global information of the full-length CDS sequence. Finally, the learned feature representations are taken as input to a dual-task module, which includes a classification task and a regression task by a parameter-sharing single-layer neural network, to predict the ribosome density distributions.

There are three advantages of our RiboMIMO method over existing prediction methods. First, unlike most of previous methods [9, 12], which only consider short sequence fragments (i.e., about 10–12 codons, roughly the length of ribosome footprints), RiboMIMO takes the full-length CDS as input. Through this way, RiboMIMO learns the contextual-aware feature representation for each codon based on the global information of the entire CDS sequence, which can thus greatly enhance the expressiveness of codon features. Second, RiboMIMO regards the ribosome density distribution of a whole gene as a training sample rather than the single ribosome density at a certain position. By regarding multiple outputs (i.e., ribosome densities of all the codons within a CDS sequence) as a whole, RiboMIMO considers the correlations between ribosome densities of adjacent codons. This multi-output setting is closer to the real situation compared with the single-output setting, because the latter generally implicitly assumes that the ribosome densities at two distinct positions are independently distributed, regardless of their distance or adjacency. Finally, the multi-input and multi-output setting enables RiboMIMO to model the contribution of any codon in a CDS sequence to the predicted ribosome density of any other codon from ribosome profiling data. Based on this modeling capacity of RiboMIMO, we also define a new metric called codon impact score (CIS) to analyze and interpret the influencing patterns between any two codons within a transcript (Fig 1C).

More detailed descriptions of our RiboMIMO framework can be found in the following sections. For the purpose of simplicity and clarity, we will ignore the subscript *g* in the remaining part of this paper.

### Sequence encoding

Codons are triplets of nucleotides (e.g., ATG) with 64 combinations consisting of A, C, G and T. Here, we use the one-hot encoding scheme to encode the codon features of mRNA sequences. More specifically, we use $z_{i}^{\left(\text{codon}\right)}\in {\left\{0,1\right\}}^{64}$ to denote the one-hot encoding of codon *x*_*i*_. However, the one-hot encoding scheme is not able to capture the editing distances among codons. For example, the editing distance between AGA and AGG is closer than that between AGA and TCG, which unfortunately cannot be represented by the one-hot encodings of codons. In addition, the relations between codons and the corresponding encoded amino acids are not considered in one-hot encoding of codons. Although the information of nucleotides and amino acids can be implicitly represented by codon encodings, it can not guarantee that these correspondences can be captured from the data. To address these issues, we introduce an additional nucleotide encoding $z_{i}^{\left(\text{nt}\right)}\in {\left\{0,1\right\}}^{4\times 3}$ and an amino acid encoding $z_{i}^{\left(\text{aa}\right)}\in {\left\{0,1\right\}}^{21}$. The nucleotide encoding is obtained by concatenating three one-hot encodings of four nucleotide types (i.e., A, T, C and G), while the amino acid encoding is derived through one-hot encodings of 20 amino acids and stop codons. Overall, the final encoding of codon *x*_*i*_ is represented by

$$\begin{array}{c} z_{i}=\left[z_{i}^{\left(\text{codon}\right)};z_{i}^{\left(\text{nt}\right)};z_{i}^{\left(\text{aa}\right)}\right]\in R^{97}, \end{array}$$ (3)

where [⋅] stands for the concatenation operator.

### Context-aware codon feature representation by deep neural networks

After the encoding module, the feature representations of codons are further embedded through a deep neural network module, which consists of a message propagation network for information gathering and distributing, and a fully connected network for further feature extraction.

Our model aims to capture the contextual patterns that are crucial for regulating the ribosome elongation rates. To achieve this goal, RiboMIMO first employs a two-layer bi-directional gated recurrent unit (Bi-GRU) network [21] to perform message propagation in both forward and backward directions along the input sequence. GRUs can memorize and extract useful information from previous inputs, and then update the current state, which allows the influence of any contextual determinants to be successfully delivered to distant locations along the sequence. The bi-directional setting further enhances the modeling of such influence. Unlike previous models [9, 12], which mainly focus on local contexts near the ribosome A site and ignore the possible influence from remote codons, here, our model takes the full-CDS sequence as input and learns a context-aware feature representation of the whole transcript for each codon. Under the RiboMIMO framework, each codon can contribute to the hidden states of GRUs through feature updating and influence the feature representations of other codons by message passing. More specifically, for a single forward directional GRU, the hidden state $h_{i}^{\left(\text{forward}\right)}$ of codon at position *i* is updated as the summation of the hidden state of its previous codon $h_{i-1}^{\left(\text{forward}\right)}$ and the output of new gate ***n***_*i*_, weighted by a update gate ***u***_*i*_. Here, the output of new gate ***n***_*i*_ is the combination of current input ***z***_*i*_ after a linear layer and the previous hidden state $h_{i-1}^{\left(\text{forward}\right)}$, whose contribution is determined by a reset gate ***r***_*i*_, followed by a hyperbolic tangent activation function. That is,

$$\begin{array}{cc} h_{i}^{\left(\text{forward}\right)}= & u_{i}*h_{i-1}^{\left(\text{forward}\right)}+\left(1-u_{i}\right)*n_{i}, \end{array}$$ (4)

$$\begin{array}{cc} n_{i}= & \text{tanh}(W_{zn}\cdot z_{i}+r_{i}*\left(W_{hn}\cdot h_{i-1}^{\left(\text{forward}\right)}\right)), \end{array}$$ (5)

$$\begin{array}{cc} r_{i}= & \sigma (W_{zr}\cdot z_{i}+W_{hr}\cdot h_{i-1}^{\left(\text{forward}\right)}), \end{array}$$ (6)

$$\begin{array}{cc} u_{i}= & \sigma (W_{zu}\cdot z_{i}+W_{hu}\cdot h_{i-1}^{\left(\text{forward}\right)}), \end{array}$$ (7)

where * stands for the Hadamard product, tanh stands for the hyperbolic tangent function and *σ* stands for the sigmoid function. Here we ignore the bias terms of the linear layers for clarity. The hidden states of the backward direction can be obtained in the similar way.

The hidden states of the Bi-GRU network are the concatenations of both forward and backward states, that is,

$$\begin{array}{c} h_{i}^{\left(\text{1}\right)}=\left[h_{i}^{\left(\text{forward}\right)};h_{i}^{\left(\text{backward}\right)}\right]. \end{array}$$ (8)

In addition, we use two layers of Bi-GRUs, in which the hidden state of the first recurrent layer $h^{\left(1\right)}=\left(h_{1}^{\left(1\right)},\ldots ,h_{n}^{\left(1\right)}\right)$ is the input to the second layer. That is,

$$\begin{array}{c} h^{\left(\text{2}\right)}=\text{Bi-GRU}\left(h^{\left(\text{1}\right)}\right). \end{array}$$ (9)

Here, the hidden states ***h***^(2)^ learned by the Bi-GRUs generally contain feature information from codons elsewhere, and need to be converted into feature representations within a common space for the down-stream tasks. This is realized by a parameter-sharing single-layer fully connected neural network with a rectified linear unit (ReLU) activation function. That is, the final context-aware feature representation of the codon at position *i* in our framework is defined as

$$\begin{array}{c} h_{i}=\text{ReLU}\left(W_{\text{fc}}\cdot h_{i}^{\left(\text{2}\right)}\right), \end{array}$$ (10)

where ***W***_fc_ stands for the learnable weight parameter for the fully connected neural network.

### The dual-task module

RiboMIMO captures the relations of translation elongation rates between neighboring codons through a dual-task objective, i.e., with a regression task and a classification task. The targets of the two tasks are both to predict the ribosome density distributions, only using discretized labels for the classification task.

Here, we discretized the ribosome densities into different classes. The distribution of ribosome densities was close to Gaussian in log scale and thus we determined the thresholds for discretization using different numbers of standard deviations derived from the plotted distribution (S2 Fig). In particular, we discretized the ribosome densities into three classes, and the thresholds of discretization are selected according to the average density (*μ*) and the average density plus two standard deviations (*μ* + 2*σ*), calculated from all the training data. The codons with ribosome densities below average (<*μ*) are labeled as “fast”, while those above average (≥*μ*) are labeled as “slow”. In addition, those codons with ribosome densities higher than *μ* + 2*σ* are regarded as “pausing”, that is,

$$\begin{array}{c} {\hat{d}}_{i}=\{\begin{array}{cc} \text{fast} & d_{i}<\mu ,\\ \text{slow} & \mu \leq d_{i}<\mu +2\sigma ,\\ \text{pausing} & \mu +2\sigma \leq d_{i}, \end{array} \end{array}$$ (11)

where ${\hat{d}}_{i}$ stands for the discretized ribosome density. Such a threshold was also used in a previous study [11]. Noted that we did not use a threshold of *μ* − 2*σ* because we care more about positions with slow ribosome elongation rates than those with fast rates.

For both regression and classification tasks in the dual-task module, RiboMIMO uses linear projections of the feature representation ***h*** to predict the ribosome density for each codon, that is,

$$y_{i}=W_{\text{reg}}\cdot h_{i},$$ (12)

$${\hat{y}}_{i}^{l}=W_{\text{cls}}^{l}\cdot h_{i},\;l\in \left\{\text{fast},\text{slow},\text{pausing}\right\},$$ (13)

where *y*_*i*_ stands for the predicted ribosome density, ${\hat{y}}_{i}^{l}$ stands for the unnormalized log-probability of discretized ribosome density with label *l*, ***W***_reg_ and $W_{\text{cls}}^{l}$ stand for the learnable weight parameters for the regression and classification task, respectively.

All the parameters of RiboMIMO are optimized by minimizing a regression loss $L^{\left(\text{reg}\right)}$ and a classification loss $L^{\left(\text{cls}\right)}$ simultaneously, that is,

$$\begin{array}{c} L=\underset{L^{\left(\text{reg}\right)}}{\underset{︸}{\left[\frac{1}{n}\sum_{i=1}^{n}{\left(d_{i}-y_{i}\right)}^{2}\right]}}+\alpha \cdot \underset{L^{\left(\text{cls}\right)}}{\underset{︸}{\left[\frac{1}{n}\sum_{i=1}^{n}(-\sum_{l}I\left({\hat{d}}_{i}=l\right)\cdot {\hat{y}}_{i}^{l}+\log\sum_{l}\exp\left({\hat{y}}_{i}^{l}\right))\right]}} \end{array}$$ (14)

where $L^{\left(\text{reg}\right)}$ stands for the mean squared error loss in the regression task, $L^{\left(\text{cls}\right)}$ stands for the cross-entropy loss in the classification task, and *α* stands for a weighting factor for balancing the two losses. We used a value of 1.0 for *α* here.

### Codon impact score

To provide the interpretability of RiboMIMO, we also define a new metric named the codon impact score (CIS) measuring the impact of codon feature at position *i* to the predicted ribosome density at position *j* within the same gene, that is,

$$\begin{array}{c} {\text{CIS}}_{i,j}=\frac{\partial y_{j}}{\partial z_{i}}, \end{array}$$ (15)

where *z*_*i*_ stands for the input of the codon feature at position *i* and *y*_*j*_ stands for the predicted ribosome density of the codon at position *j* by RiboMIMO. Note that here the CIS is defined as the first order partial derivatives of individual output variables with respect to individual input variables, which has been popularly used in the computer vision community [22]. Here the CIS can also be regarded as the first order estimation of the Taylor expansion of our model, that is,

$$\begin{array}{c} y_{j}=\sum_{i}\frac{\partial y_{j}}{\partial z_{i}}\cdot z_{i}+o\left(z\right)\approx \sum_{i}{\text{CIS}}_{i,j}\cdot z_{i}, \end{array}$$ (16)

where *o*(*z*) stands for an infinitesimal of higher order. In this view, the ribosome density can be estimated by computing the accumulation effect of CIS values along the CDS region, and the CIS of each input codon thus measured its corresponding contribution to the prediction. Basically, the CIS can be used to measure the impact of a codon of interest to the predicted density of any other codon within the same gene regardless of the distance and direction. This is also enabled by the particular end-to-end design of the RiboMIMO architecture in which an input variable can contribute to any output variable. The CIS-related analyses in this paper were performed using the same trained model for each dataset.

## Results

### RiboMIMO accurately predicts ribosome densities from full-length CDS sequences

We tested RiboMIMO on four datasets from the *E.coli* and *S.cerevisiae* species, respectively. For each dataset, we performed a cross-validation procedure by randomly splitting the genes into 10 folds to assess the generalization capacity of our method. In each fold, one subset of the data was held out as test data while the remaining nine folds were used as training data. We left out 1/10 of training data as the validation set for determining the early stop criterion. To evaluate the performance of our model, we used the Pearson’s correlation coefficients to measure the correlations between the predicted ribosome densities vs. the experimentally measured values. We also calculated the gene-wise correlations by collecting the predicted and measured ribosome densities for individual genes in the test set at each cross-validation. The prediction results of several example genes can be found in S3 Fig.

We first compared our method with other ribosome density prediction approaches, including a statistical based method RUST [9], a wavelet decomposition based method riboShape [10] and a machine learning based method iXnos [12]. Our comparison results showed that RiboMIMO can significantly outperform the state-of-the-art baseline methods, with an increase of 5.79%, 7.60%, 3.73% and 3.64% compared to the best baseline iXnos upon gene-wise correlations on the Mohammad16, Subtelny14, Mohammad19-1 and Mohammad19-2 datasets, respectively (Table 1 and S4 Fig).

**Table 1:** Performance evaluation of different prediction methods, measured in terms of the Pearson’s correlation coefficient.

Methods	Dataset			
	Mohammad16	Subtelny14	Mohammad19-1	Mohammad19-2
riboShape	0.4308 ± 0.1117 (****)	0.3892 ± 0.0993 (****)	0.3312 ± 0.1299 (****)	0.2879 ± 0.1160 (****)
RUST	0.5700 ± 0.1279 (****)	0.4151 ± 0.1047 (****)	0.5622 ± 0.1343 (****)	0.5775 ± 0.1308 (****)
iXnos	0.6325 ± 0.1084 (****)	0.5625 ± 0.1008 (****)	0.6333 ± 0.1282 (****)	0.6573 ± 0.1271 (****)
RiboMIMO	**0.6904 ± 0.1112**	**0.6385 ± 0.0997**	**0.6706 ± 0.1353**	**0.6937 ± 0.1345**
w/o nt & aa	0.6750 ± 0.1142 (****)	0.6312 ± 0.1021 (****)	0.6674 ± 0.1342 (****)	0.6885 ± 0.1349 (****)
w/o nt	0.6892 ± 0.1126 (**)	0.6351 ± 0.1007 (****)	0.6683 ± 0.1318 (****)	0.6897 ± 0.1352 (****)
w/o aa	0.6867 ± 0.1142 (****)	0.6345 ± 0.1014 (****)	0.6706 ± 0.1339 (ns)	0.6935 ± 0.1353 (ns)
w/o dual-task	0.6791 ± 0.1135 (****)	0.6263 ± 0.1005 (****)	0.6651 ± 0.1348 (****)	0.6833 ± 0.1346 (****)

10-fold cross-validation through randomly splitting genes into training and test sets was performed on the four datasets to assess the performance of each method. The gene-wise correlations were computed by comparing the predicted and measured ribosome densities for individual genes in the test set at each 10-fold cross-validation. The mean ± SD of the gene-wise Pearson’s correlation coefficients across the whole dataset are shown. Significant levels from Friedman tests with Dunn’s multiple comparison correction are shown in the parentheses. The statistical tests were conducted by comparing different baseline methods or the RiboMIMO model without certain modules with the original RiboMIMO model.

****: *P* < 0.0001,

***: *P* < 0.001,

**: *P* < 0.01,

*: *P* < 0.05,

ns: not significant. nt and aa stand for nucleotide encoding and amino acid encoding, respectively. The best results are shown in bold.

In the above comparison results, all the baseline methods were trained and evaluated with the same random train/test splitting fashion. For riboShape, we selected the component of wavelet decomposition with the highest similarity with the original data, and used the results of the asymmetry kernel scheme, which was the best among the three methods proposed in the original paper [10]. For RUST and iXnos, we used the input sequences with a window size of 12 mainly because this selection covered the whole ribosome footprints and it was shown to be an optimal choice in the original papers. We used the best hyperparameter settings as mentioned in the original papers, such as learning rate, optimizer, dropout rate and gradient clipping. For RiboMIMO, we implemented the framework using PyTorch [23], a popularly used open-source machine learning library. The detailed settings of the architecture and hyperparameters can be found in S2 Table.

We also performed ablation studies on the feature encoding and dual-task training to validate the contributions of the two modules to the performance of ribosome density prediction. We first removed the nucleotide encoding and the amino acid encoding (i.e., using only codon encoding) and retrained our model (denoted as “w/o nt & aa”). The results of removing either nucleotide encoding (denoted as “w/o nt”) or amino acid encoding (denoted as “w/o aa”) were also evaluated. As shown in Table 1, we observed a significant decrease of 1.54%, 0.73%, 0.32% and 0.52% upon gene-wise correlation without nucleotide encoding and amino acid encoding on the Mohammad16, Subtelny14, Mohammad19-1 and Mohammad19-2 datasets, respectively. The performance without either nucleotide encoding or amino acid encoding also decreased to some extent, indicating the necessarity of incorporating all the encodings. We also examined the results after removing the classification task in the dual-task module and using only the regression loss for training (denoted as “w/o dual-task”). We observed a decrease of 1.13%, 1.22%, 0.55% and 1.04% upon gene-wise correlation without dual-task training on the four datasets, respectively. Thus, the classification task in our dual-task module can improve the performance of the regression task. Here, the introduction of the classification task can be thought as a kind of multi-task learning. Optimizing diverse but correlated tasks simultaneously could improve their performance compared with learning them individually. More specifically, in the regression task, the labels of ribosome densities were normalized within each gene. Here, the ribosome densities from different genes may exhibit diverse distributions. On the other hand, for the labels in the classification task, though they were discretized from the same labels in the regression tasks, the discretization thresholds were obtained by referring to the distributions of all the genes in the training set, i.e., with the consideration of global information of the whole training dataset. In addition, the classification loss may bring a regularization effect as both tasks (i.e., regression and classification tasks) took the same feature representations as input and updated the shared parameters through back-propagation. Therefore, in principle, the performance of the regression task can be improved by introducing the classification loss.

More results on the robustness of our model to different evaluation metrics, genomic/sequencing factors, sequence similarities, hyperparameters, and cross-dataset evaluation can be found in the supplementary information (S1 Text).

### RiboMIMO detects the contributions of remote codons to the predicted ribosome density of current A site

One major improvement of RiboMIMO over previous prediction methods is that it can model the ribosome density distribution at full-length CDS level. Most of the previous computational models focus on local sequence features centered at the ribosomal A sites with a length of about 10–12 codons or slightly higher, and ignored the remote influence. In this section, we demonstrated, for the first time (to the best of our knowledge), how input lengths influence the ribosome density predictions. In particular, we truncated the whole CDS into fragments with different lengths and fed them into the RiboMIMO model for training and then compared the corresponding performance with that of the original full-length input setting. Noted that RiboMIMO can take input with arbitrary lengths and make prediction for each codon position. Thus, the truncated sequences can be directly used as training data without modifying the model architecture. We trained the RiboMIMO models separately using the truncated sequences with lengths ranging from 8 to 128 codons, and evaluated their performances using full-length coding sequences as test data (Table 2). We observed that with the increase of the lengths of the input sequences, the prediction performance significantly improved and reached the highest when taking the full-length CDS as input. This result proved that the remote codons may have the potential to influence the ribosome densities, and modeling the translation elongation speed in a full-length CDS scheme can be of great benefit to the ribosome density prediction.

**Table 2:** Performance evaluation of RiboMIMO with different CDS lengths as input, measured in terms of Pearson’s correlation coefficient.

Input CDS length	Dataset			
	Mohammad16	Subtelny14	Mohammad19-1	Mohammad19-2
8	0.6553 ± 0.1030	0.5682 ± 0.0987	0.6209 ± 0.1400	0.6656 ± 0.1349
16	0.6724 ± 0.1058 (****)	0.5858 ± 0.0969 (****)	0.6275 ± 0.1397 (****)	0.6765 ± 0.1355 (****)
32	0.6837 ± 0.1096 (****)	0.5985 ± 0.0965 (****)	0.6352 ± 0.1383 (****)	0.6812 ± 0.1353 (****)
64	0.6903 ± 0.1101 (ns)	0.6147 ± 0.0994 (****)	0.6529 ± 0.1314 (****)	0.6855 ± 0.1359 (****)
128	0.6905 ± 0.1073 (ns)	0.6289 ± 0.0994 (****)	0.6613 ± 0.1346 (****)	0.6882 ± 0.1355 (****)
full	0.6938 ± 0.1100 (ns)	0.6385 ± 0.0997 (****)	0.6706 ± 0.1353 (****)	0.6937 ± 0.1345 (****)

10-fold cross-validation through randomly splitting genes into training and test sets was performed on the four datasets to assess the performance of the model with each input CDS length. The gene-wise correlations were computed by comparing the predicted and measured ribosome densities for individual genes in the test set at each 10-fold cross-validation. The mean ± SD of the gene-wise Pearson’s correlation coefficients across the whole dataset are shown. Significant levels from Friedman tests with Dunn’s multiple comparison correction are shown in the parentheses. The statistical tests were conducted by comparing each group (e.g., with input CDS length of 16) with the previous group (e.g., with input CDS length of 8).

****: *P* < 0.0001,

***: *P* < 0.001,

**: *P* < 0.01,

*: *P* < 0.05,

ns: not significant.

To further reveal how the sequence patterns of remote codons contribute to the predicted ribosome density of a specific codon, we compared the performance of our models with and without using the input features from the remote codon regions. More specifically, we separated the CDS sequence into three regions named “center”, “left” and “right” based on their relative positions with respect to the ribosomal A site (Fig 1D). The “center” region (with a length of 10 codons ranging from positions −5 to + 4 around the A site) is generally regarded the most crucial for ribosomal A site decoding. Here, we further evaluated the contributions of the remaining parts of the CDS sequence, namely “left” region (from start codon to position −6 at 5′ end) and “right” region (from position + 5 to stop codon at 3′ end), by measuring the performance after removing these specific regions as input. We observed that the remote regions of both “left” and “right” regions can indeed affect the prediction performance of ribosome densities at the A sites, with a decrease of 5.57%, 2.90%, 6.37% and 5.44% in the Pearson’s correlation coefficients on the four datasets, respectively (Table 3). Therefore, the codons located at remote positions can influence and contribute to the prediction of ribosome densities, indicating the necessity and superiority of modeling ribosome density distributions at full-CDS level. Interestingly, codons at the “left” regions seemed to display a more important role in ribosome density prediction, with a decrease of performance by about 3.78%, 2.17%, 5.37% and 5.22% for the four datasets, respectively, compared to those codons at the “right” regions, with a decrease of performance by about 1.57%, 0.17%, 0.26% and 0.05% for the four datasets, respectively. Such an observation was in accordance with the fact that mRNAs are read from the 5′ to 3′ direction by ribosomes [24]. The effects of distant codons on the predicted ribosome density may be explained by the accumulated effect of the translation process, such as the overall dwell time spent in the previous steps during elongation, which may activate the mRNA degradation mechanism [25] and thus influence the ribosome distribution of the successive part of the sequence.

**Table 3:** Performance evaluation of RiboMIMO with different CDS regions as input, measured in terms of Pearson’s correlation coefficient.

Input CDS region	Dataset			
	Mohammad16	Subtelny14	Mohammad19-1	Mohammad19-2
center+left+right	0.6938 ± 0.1100	0.6385 ± 0.0997	0.6706 ± 0.1353	0.6937 ± 0.1345
center+left	0.6781 ± 0.1131 (****)	0.6368 ± 0.1010 (*)	0.6680 ± 0.1357 (***)	0.6932 ± 0.1343 (ns)
center+right	0.6560 ± 0.1127 (****)	0.6168 ± 0.1016 (****)	0.6169 ± 0.1361 (****)	0.6415 ± 0.1315 (****)
center	0.6381 ± 0.1165 (****)	0.6095 ± 0.1035 (****)	0.6069 ± 0.1402 (****)	0.6393 ± 0.1313 (****)

10-fold cross-validation through randomly splitting genes into training and test sets was performed on the four datasets to assess the performance of each input region. The gene-wise correlations were computed by comparing the predicted and measured ribosome densities for individual genes in the test set at each 10-fold cross-validation. The mean ± SD of the gene-wise Pearson’s correlation coefficients across the whole dataset are shown. Significant levels from Friedman tests with Dunn’s multiple comparison correction are shown in the parentheses. The statistical tests were conducted by comparing the RiboMIMO models using parts of the CDS regions as input with that using full-length CDS regions. The notations of CDS regions (i.e., “center”, “left” and “right”) are defined in both Fig 1D and main text.

****: *P* < 0.0001,

***: *P* < 0.001,

**: *P* < 0.01,

*: *P* < 0.05,

ns: not significant.

### RiboMIMO extracts the distance related patterns for predicting ribosome densities

To quantify the pair-wise contribution from the input codon at position *i* to the predicted ribosome density of the output codon at position *j*, we calculated the codon impact score CIS_*i*,*j*_ for codon pair (*x*_*i*_, *x*_*j*_) as defined in Eq (15). The absolute value of a CIS refers to the strength of the corresponding relation, while the sign indicates whether the input codon positively or negatively influences the predicted ribosome density of the output codon. The CIS values can be considered the interpreters of the trained RiboMIMO model and thus provide the underlying contextual patterns and rules for understanding the translation elongation process.

We first calculated the contributions of distances for the predicted ribosome densities. Suppose that the relative distance of a codon pair (*x*_*i*_, *x*_*j*_) is denoted by *d* = *i* − *j*. Then the contribution CIS_*d*_ for distance *d* is defined as,

$$\begin{array}{c} {\text{CIS}}_{d}=\frac{\sum_{i,j}\left|{\text{CIS}}_{i,j}\right|\cdot I\left(i-j=d\right)}{\sum_{i,j}I\left(i-j=d\right)}. \end{array}$$ (17)

Here, a CIS_*d*_ value with a positive *d* refers to the contribution from the 3′ direction, while a CIS_*d*_ value with a negative *d* refers to that from the 5′ direction.

**Fig 2:**
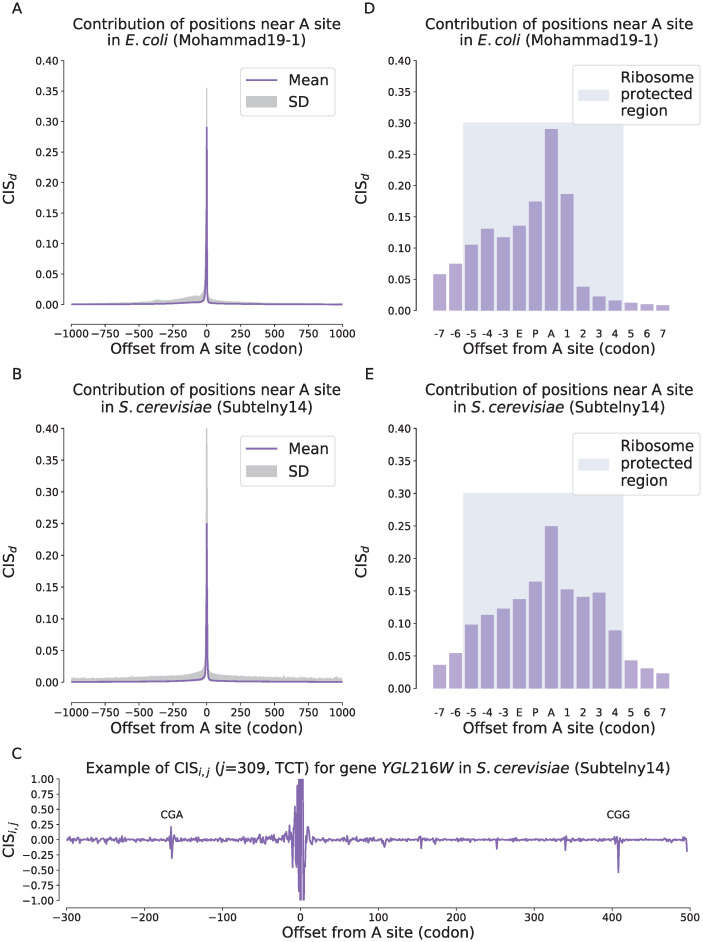
Contributions of the codon features at different distances to the predicted ribosome densities measured by the codon impact score (CIS) on the Mohammad19-1 (*E.coli*) (A and D) and the Subtelny14 (*S.cerevisiae*) (B and E) datasets. (A–B). The average contributions of input codons to the predicted ribosome density of current codon with respect to the relative distances on the Mohammad19-1 (A) and the Subtelny14 (B) datasets. The horizontal axis stands for the offset from the input codon to the A site of current codon, where a positive value stands for the 3′ direction while a negative value stands for the 5′ direction. The solid line refers to the average CIS_*d*_ values while the shaded region stands for the scope of one standard deviation. (C). Case study for gene *YGL216W* in *S.cerevisiae* of the Subtelny14 dataset, in which the CIS_*i*,*j*_ values of different positions with respect to the predicted ribosome density of the codon at position *j* = 309 are shown. The horizontal axis is the offset of the codon at position *i* relative to the codon at position *j* = 309. (D–E). The zoomed-in contributions of those codons near the ribosomal A sites on the Mohammad19-1 (D) and the Subtelny14 (E) dataset. The blue shaded regions in (D) and (E) stand for the ribosome protected regions of the mRNA sequence.

We observed a significantly high enrichment of the contributions at those codons near A sites on both *E.coli* and yeast datasets (Fig 2). This was consistent with the previous finding that the codon features around the A site generally dominate the prediction of ribosome density [12]. Although the contributions shrunk along with the distances at both directions, we observed that those codons located far from the ribosomal A site can still influence the predicted ribosome density through a long distance (Fig 2C and the shaded regions of SD in Fig 2A and 2B and S5A and S5B Fig). The long-tailed distribution of the contributions from long distances suggested that although such a kind of remote influence was relatively rare, it indeed existed. This phenomenon was also consistent with the results shown in the previous section that the prediction performance of ribosome densities can be improved by increasing the input CDS length.

Next, we focused on the regions near the ribosomal A sites (i.e., ribosome protected regions), to further examine the contributions of individual positions at single-codon resolution (Fig 2D and 2E and S5C and S5D Fig). The ribosomal A site has been known as the most crucial position in determining the ribosome densities, where the tRNA is recruited and recognized. The time required for selecting a correct tRNA during A site decoding greatly contributes to the overall translation elongation speed [2]. We observed that the contribution of the A site codon was the highest among all the positions on the Subtelny14 dataset of yeast, while the other codons inside the ribosome protected region also played an important role in predicting ribosome densities (Fig 2E). Similar patterns were also observed on two Mohammad19 datasets of *E.coli* (Fig 2D and S5D Fig). However, on the Mohammad16 dataset, the codon at the E site seemed to be more important than that at the A site and there was a valley at the center of the ribosome protected region (S5B Fig). One possible explanation was that, during cell lysis of ribosome profiling experiments of the Mohammad16 dataset, chloramphenicol (Cm) is added to the lysis buffer [13], which has been shown to cause the E site pausing [14]. By using the optimized pre-treatment and filtering protocols, the E site signals observed on the Mohammad16 dataset did not appear on the two Mohammad19 datasets, indicating that these signals could be due to the artifact caused by the experimental bias.

### RiboMIMO identifies the factors affecting translation elongation rates at both codon and amino acid levels

Codon usage bias has been widely believed to be one of the major determinants of translation efficiency [26]. A rare codon generally means a low abundance level of the corresponding aminoacyl-tRNA (i.e., the corresponding amino acid loaded tRNA). Thus more likely it will cost more time for the ribosome to upload the correct amino acid. We used the codon adaptation index (CAI) [27] to measure the relative codon usage bias from endogenous genes. A codon with a higher CAI refers to a higher usage frequency compared to its synonymous codons encoding the same amino acid, while a codon with a lower CAI refers to a rare one, which is more likely to cause a slow elongation rate. Therefore, codon usage bias scores are supposed to correlate with the ribosome densities. tRNA adaptation index (tAI) is another measure of the decoding rate of translation elongation, which is also supposed to associate with the ribosome densities. Other biological factors that are likely to have associations with the translation elongation rates were also included in our analyses, such as GC content, local folding energy and amino acid hydrophobicity.

Here, the Mohammad19-1 and the Mohammad19-2 datasets were biological replicates, therefore we only selected the Mohammad19-1 and the Subtelny14 datasets for our analyses. The CAI value for each codon type was calculated using all the genes in the datasets, according to its definition [27]. The tAI value for each codon type was collected from [4]. The GC content for each codon was computed directly. The folding energies of mRNA sequences with a sliding window of 60 nucleotides (i.e., 20 codons) were computed using the RNAfold software in the ViennaRNA package [28]. The hydrophobicity scales were obtained from [29]. The ribosome densities were predicted using the RiboMIMO model and then averaged for each codon type. To investigate the impacts of single codon types on translation elongation dynamics learned by RiboMIMO, we also defined a new metric for each codon type *c*, denoted by CIS_*c*,⋅_, that is,

$$\begin{array}{c} {\text{CIS}}_{c,\cdot }=\frac{\sum_{i,j}\left|{\text{CIS}}_{i,j}\right|\cdot I\left(x_{i}=c\right)}{\sum_{i,j}I\left(x_{i}=c\right)}. \end{array}$$ (18)

**Fig 3:**
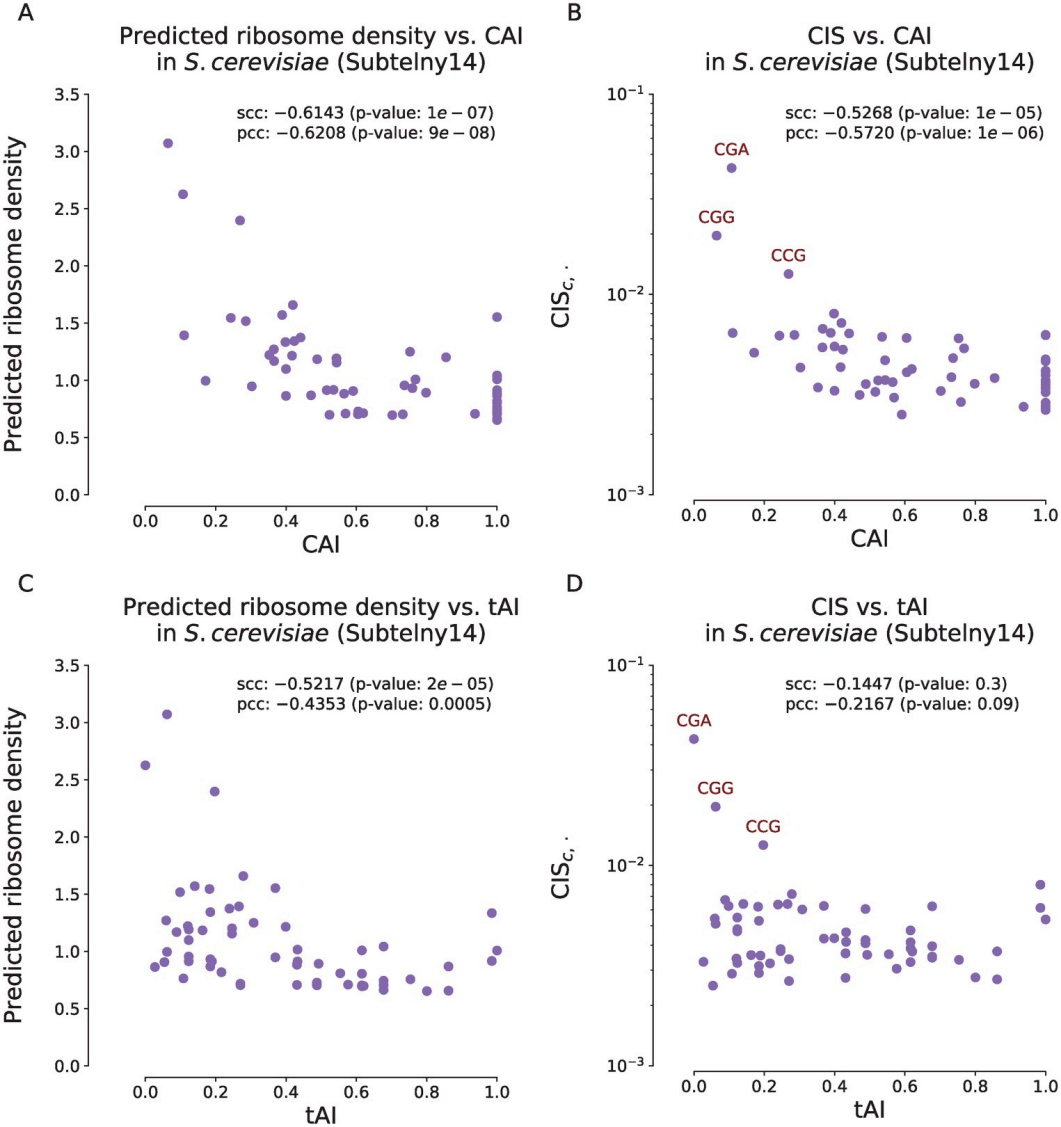
Correlations of ribosome densities and codon impact scores CIS_*c*,⋅_ at the A sites with codon adaptation index (CAI) (A–B) and tRNA adaptation index (tAI) (C–D) for the Subtelny14 dataset. (A). Scatter plots of averaged predicted ribosome densities and CAI scores for individual codon types. (B). Scatter plots of CIS_*c*,⋅_ values in log scale and CAI scores for individual codon types. (C). Scatter plots of averaged predicted ribosome densities and tAI scores for individual codon types. (D). Scatter plots of CIS_*c*,⋅_ values in log scale and tAI scores for individual codon types. scc and pcc stand for the Spearman’s correlation coefficient and the Pearson’s correlation coefficient, respectively. In panel (B) and (D), the rare codons CGA, CGG and CCG are labeled in red.

Our analysis results demonstrated that there existed significant correlations of the codon usage bias with both ribosome densities and CIS_*c*,⋅_ values derived from RiboMIMO (Fig 3) on the Subtelny14 dataset in yeast, suggesting that our RiboMIMO framework was able to extract the codon-level characteristics for predicting translation elongation rates. More specifically, we observed that CGA, CGG and CCG were among the top three codon types with the highest CIS_*c*,⋅_ values and relatively low CAI or tAI values, indicating that our approach was able to capture the influence of rare codons. In particular, we observed that CGA was the codon with the highest CIS_*c*,⋅_ score among all the rare codons, which was consistent with the previous studies that this codon has a strong inhibitory effect in controlling translation efficiency in yeast [30].

We noticed that the associations of the CAI or tAI with the ribosome densities in *E.coli* were weak (S6A, S7A and S7C Figs), which is consistent with the previous literatures [31, 32]. Although another study observed a certain level of correlation between the decoding time and tAI in *E.coli* [33], we suggested that this could be due to the bias between different experiments in ribo-seq and tAI measurement. In S6B Fig, we observed that six codon types (TCA, TCG, TCC, TCT, AGT and AGC) with the highest CIS_*c*,⋅_ values were all serine encoding codons. Serine has been reported as a toxic amino acid for bacterias, and the levels of the corresponding aminoacyl-tRNAs are the lowest (<10%) among all tRNA types due to the competition with L-serine-deaminase [34]. This was also probably caused by the same reason previously mentioned in the last section that the *E.coli* dataset may bias towards the E site pausing resulted from chloramphenicol treatment [14]. Note that all six serine encoding codons had high CIS_*c*,⋅_ values regardless of codon usage bias, suggesting that the RiboMIMO framework can extract additional determinants of ribosome densities other than codon usage bias at amino acid level.

For the associations with other biophysical factors, we observed positive correlations of ribosome densities and CIS with GC content in both datasets (S7E–S7H Fig), which suggested that the codons with higher GC content tended to have higher ribosome densities and contributed more to the ribosome density prediction. We also observed that the CIS values obtained by our model were weakly and negatively correlated with the folding energies (S7J and S7L Fig), and the sequences with lower folding energies (i.e., more stable secondary structures) were more likely to have influence on the predicted ribosome densities. This was consistent with the known fact that the secondary structures of mRNA can reduce the elongation rates [35]. We noticed that a higher GC content may also be associated with a lower folding energy (with Spearman’s correlation −0.13 in our datasets). Therefore the influence of these two factors (i.e., GC content of the codon and local folding energy) may be correlated with each other to some extent. We also observed that the CIS was negatively correlated with the amino acid hydrophobicity in yeast though no correlation was observed in *E.coli* (S7M–S7P Fig). This result was also consistent with the previous study showing that a higher hydrophobicity is usually correlated with a higher elongation rate [36].

In summary, our RiboMIMO model and the defined codon impact score (CIS) can successfully detect the meaningful influencing factors that are associated with the translation elongation rates at both codon and amino acid levels.

### RiboMIMO captures combinatorial effects of codon and position information

To investigate the combinatorial contributions of both codon and position information, we also define the codon impact scores CIS_*c*,*d*_ for codon type *c* located at distance *d* from the ribosomal A site, that is,

$$\begin{array}{c} {\text{CIS}}_{c,d}=\frac{\sum_{i,j}{\text{CIS}}_{i,j}\cdot I\left(x_{i}=c\right)\cdot I\left(i-j=d\right)}{\sum_{i,j}I\left(x_{i}=c\right)\cdot I\left(i-j=d\right)}. \end{array}$$ (19)

**Fig 4:**
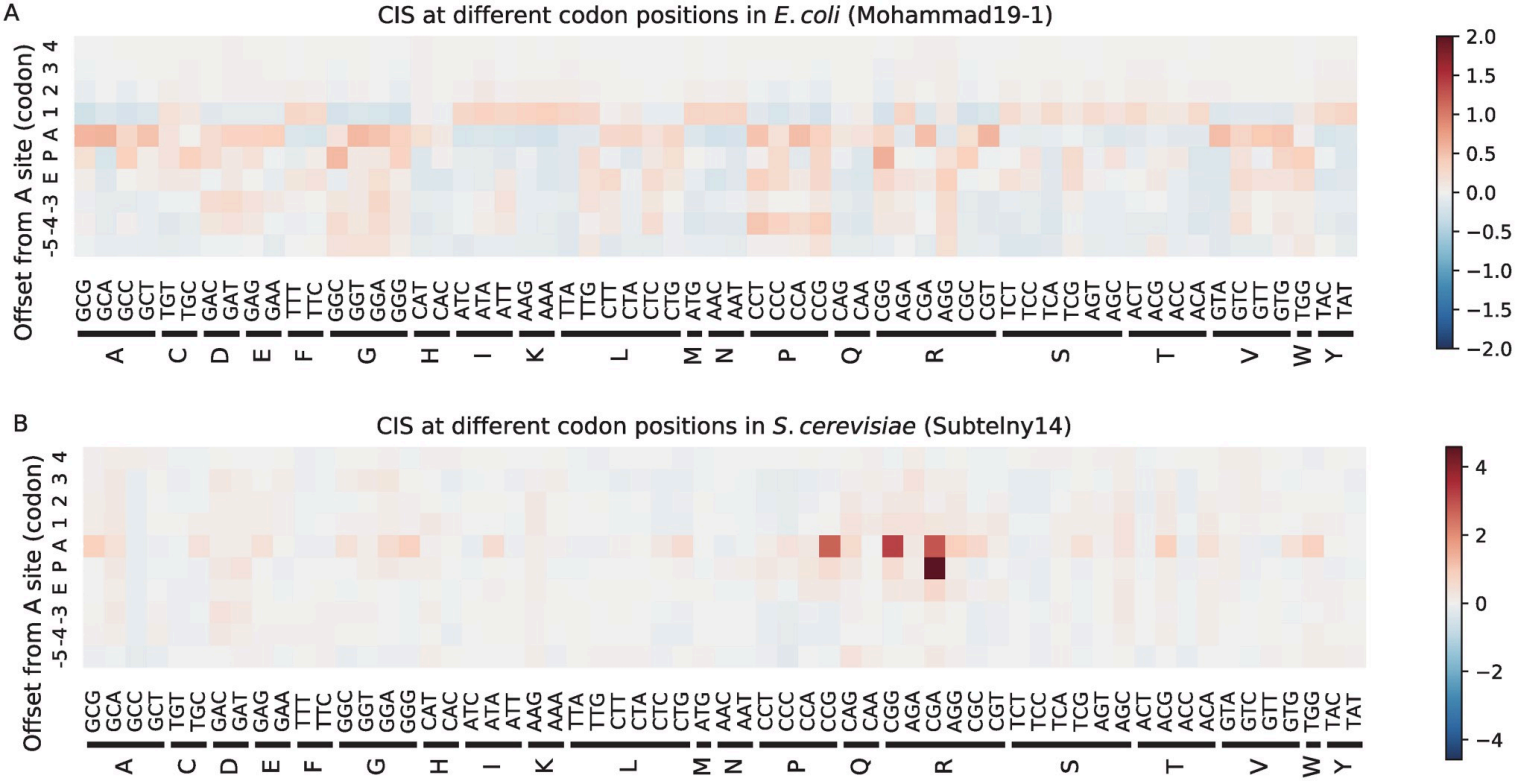
The codon-distance impact score (CIS_*c*,*d*_) values measuring the contributions of codon type *c* at distance *d* to the predicted ribosome densities of the current A site for the Mohammad19-1 (A) and Subtelny14 (B) datasets, respectively. The horizontal axes represent the codon types and vertical axes represent the positions relative to the ribosomal A sites. Only positions within the ribosome protected region (i.e., ranging from −5 to + 4 codons relative to the A sites) are shown. The positive values (red) of CIS_*c*,*d*_ refer to inhibition in translation efficiency, while the negative values (blue) refer to promotion.

For the Subtelny14 dataset of *S.cerevisiae*, we noticed that the rare codons including CGA, CGG and CCG had significant enrichment of the CIS values in the A sites. The CGA codon was also observed to have a great effect on the predicted ribosome densities at the P sites (Fig 4B). These findings agreed well with the previous results [12] that these three rare codons play important roles in predicting ribosome densities at the A sites. The phenomenon that the CGA codons at the P sites greatly influenced the ribosome elongation rates may be explained by the potential I-A wobble base paring which possibly slows down the translation speed [30].

For the Mohammad16 dataset of *E.coli*, we observed high impact scores of inhibition at the ribosomal E sites for all the codons encoding the three amino acids including serine (S), glycine (G) and alanine (A) (S8A Fig). This pattern was consistent with the previous results that the ribosome densities for these three amino acids at the E sites are generally higher than those at the A sites [14]. In particular, these three amino acids all have small side chains (−CH_2_OH for serine, −H for glycine and −CH_3_ for alanine), which may allow the binding of chloramphenicol (an elongation inhibitor added in the lysis buffer in the current ribosome profiling protocol [37]) much easier to the active site of the ribosome [38]. The two Mohammad19 datasets of *E.coli*, in comparison, were not treated with chloramphenicol and were directly processed using flash-freezing, and thus should not be biased by the experimental protocols. We observed that the signals of serine (S), glycine (G) and Alanine (A) at the E sites in the Mohammad16 dataset did not appear in the Mohammad19 datasets (Fig 4A and S9B Fig), which suggested that these signals were probably due to the bias introduced by experimental protocols and our new findings on the improved datasets should provide convincing and useful insights toward understanding the translation elongation dynamics. This finding suggested that the RiboMIMO framework can capture the unusual behaviors hidden in the measured ribosome densities, and thus provide a useful tool for analyzing ribosome profiling data.

We further defined the codon impact scores CIS_aa,*d*_ for amino acid species aa located at distance *d* from the ribosomal A site, that is,

$$\begin{array}{c} {\text{CIS}}_{\text{aa},d}=\frac{\sum_{i,j}{\text{CIS}}_{i,j}\cdot I\left(x_{i}=\text{aa}\right)\cdot I\left(i-j=d\right)}{\sum_{i,j}I\left(x_{i}=\text{aa}\right)\cdot I\left(i-j=d\right)}. \end{array}$$ (20)

As shown in S8C–S8F Fig, the amino acid level CIS could provide a closer look of the contribution landscapes associated with each amino acid species. In addition to the proline (P) and arginine (R) highlighted on the Subtelny14 dataset, tryptophan (W) also had high influence on the ribosome densities (S8D Fig). This may be explained by the fact that TGG is the only codon encoding tryptophan, while other amino acids generally have multiple synonymous codons, most of which are associated with faster elongation rates.

In a word, the above analysis results derived by RiboMIMO were consistent with the previous findings in the literatures, indicating the robustness and reliability of our prediction results.

### RiboMIMO provides new insights into translation elongation dynamics affected by pairs of adjacent and distant codons

Previous research showed that certain pairs of adjacent codons can inhibit the translation efficiency, in which the paired effects can be distinguished from the effects of single codons [39]. Through design of random pairs of adjacent codons for green fluorescent protein (GFP) genes, 17 adjacent codon pairs were identified from over 35,000 GFP variants to associate with expression reduction [39], as summarized in S3 Table. To test whether our model can also learn such an effect from ribosome profiling data, we defined the codon impact score ${\text{CIS}}_{c_{1},c_{2}}^{\left(\text{adj}\right)}$ for each pair of adjacent codons (*c*_1_, *c*_2_), that is,

$$\begin{array}{c} {\text{CIS}}_{c_{1},c_{2}}^{\left(\text{adj}\right)}=\frac{\sum_{i,j}\left(\right|{\text{CIS}}_{i,j}+{\text{CIS}}_{i+1,j}\left|\right)\cdot I\left(x_{i}=c_{1}\right)\cdot I\left(x_{i+1}=c_{2}\right)}{\sum_{i,j}\cdot I\left(x_{i}=c_{1}\right)\cdot I\left(x_{i+1}=c_{2}\right)}. \end{array}$$ (21)

We also defined the codon impact score ${\text{CIS}}_{c}^{\left(\text{single}\right)}$ measuring the marginal contribution of a single codon *c*, that is,

$$\begin{array}{c} {\text{CIS}}_{c}^{\left(\text{single}\right)}=\frac{\sum_{i,j}\left(\right|{\text{CIS}}_{i,j}\left|\right)\cdot I\left(x_{i}=c\right)}{\sum_{i,j}\cdot I\left(x_{i}=c\right)}. \end{array}$$ (22)

We obtained an AUROC (area under receiver operating characteristic curve) of 0.985 using ${\text{CIS}}_{c_{1},c_{2}}^{\left(\text{adj}\right)}$, and an AUROC of 0.962 using ${\text{CIS}}_{c_{1}}^{\left(\text{single}\right)}\cdot {\text{CIS}}_{c_{2}}^{\left(\text{single}\right)}$. Noted that the AUROC score can be less informative when the numbers of positive and negative samples are far from equal. In fact, there were 17 positive pairs and 3704 negative ones in this case, resulting in a skewed positive to negative ratio of 0.0046. Therefore, we focused more on the AUPR (area under precision recall curve) score for each CIS, which should provide a better metric for evaluating in such an imbalanced dataset. We obtained an AUPR score of 0.444 for ${\text{CIS}}_{c_{1},c_{2}}^{\left(\text{adj}\right)}$ and 0.248 for ${\text{CIS}}_{c_{1}}^{\left(\text{single}\right)}\cdot {\text{CIS}}_{c_{2}}^{\left(\text{single}\right)}$. These AUPR results showed that although the marginal effect may play an important role in calculating the contributions of adjacent codons, there were complicated feature patterns that cannot be simply reflected by single codons.

As a result, our model can extract the paired contributions of adjacent codon pairs more than just the simple accumulation of their marginal contributions from the data. Although the contributions of paring codons learned by our model seemed to be symmetric, there existed several non-symmetric contributions. For example, the contributions of GTA-CCG and CTC-CCG were strong, while those of CCG-GTA and CCG-CTC were relatively weak. In other words, the contributions would be stronger when the rare codon CCG is located at the 3′ end. These results could provide insightful hints into understanding the translation elongation dynamics.

In the previous sections, we showed that RiboMIMO was able to reveal that remote codons can contribute to the predicted ribosome densities of current A sites. To further illustrate how such a remote impact can be learned by RiboMIMO, we also defined an impact score ${\text{CIS}}_{c_{1},c_{2}}^{\left(\text{remote}\right)}$ for those donor-acceptor codon pairs (*c*_1_, *c*_2_) with long-range associations, i.e., with a distance >20 codons, that is,

$$\begin{array}{c} {\text{CIS}}_{c_{1},c_{2}}^{\left(\text{remote}\right)}=\frac{\sum_{i,j}{\text{CIS}}_{i,j}\cdot I\left(x_{i}=c_{1}\right)\cdot I\left(x_{j}=c_{2}\right)\cdot I(|i-j|>20)}{\sum_{i,j}I\left(x_{i}=c_{1}\right)\cdot I\left(x_{j}=c_{2}\right)\cdot I(|i-j|>20)}. \end{array}$$ (23)

The term “donor” indicated the codon position that contributed to the ribosome density prediction of other positions. The term “acceptor” indicated the codon position where the ribosome density is predicted. In other words, the “donor” codon *c*_1_ had certain impacts or associations on the ribosome density at the “acceptor” codon *c*_2_.

**Fig 5:**
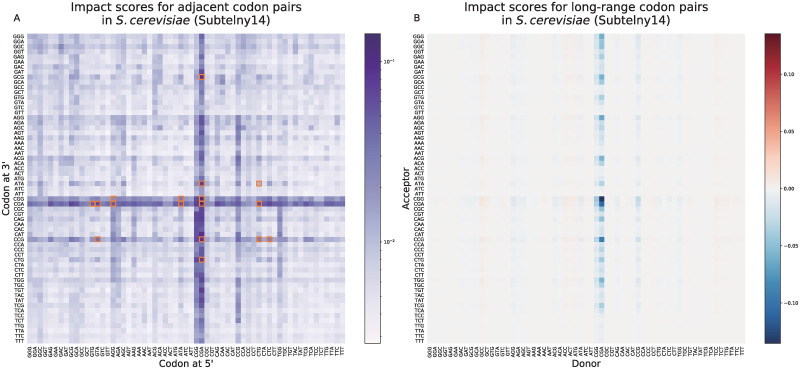
The analysis results on the impacts of adjacent codon pairs (A) and long-range codon pairs (B) on the predicted ribosome densities for the Subtelny14 dataset. (A). Impact scores of adjacent codon pairs (${\text{CIS}}_{c_{1},c_{2}}^{\left(\text{adj}\right)}$) showed in log scale. Orange boxes show 17 codon pairs reported to have influence on the protein expression levels in yeast [39]. (B). Impact scores of long-range codon pairs (${\text{CIS}}_{c_{1},c_{2}}^{\left(\text{remote}\right)}$) in log scale. The impact scores are asymmetric, in which the influencing directions are from donors to acceptors. The ${\text{CIS}}_{c_{1},c_{2}}^{\left(\text{remote}\right)}$ values were calculated using the CIS values as defined in Eq (23).

We observed a strong impact from the CGA codon (Fig 5B), which was discovered in the previous study to be the only codon in yeast decoded by the I-A wobble base pairing [40]. The CGA codon is more likely to cause ribosome stalling during the translation process, waiting for the complementarity pairing of tRNA [41], which thus may affect the translation efficiency of the transcript. To further reveal the associations behind the codon pairs with long-range influence, we particularly looked into two groups, i.e., those with the long-range donor-acceptor pairs with |CIS| ≤ 0.01 (served as a negative control) and |CIS| > 0.2, and then plotted the predicted ribosome densities for each group (S10 Fig). We observed that the distribution of group |CIS| > 0.2 was greatly different from that of the control group |CIS| ≤ 0.01 (*P* < 0.0001 with *t* test). More specifically, the ribosome densities of the donors and acceptors in the |CIS| > 0.2 group were both higher than those of the control group and were negatively correlated, with a Pearson’s correlation of −0.27 (and a Spearman’s correlation −0.43), while the Pearson’s correlation for |CIS| ≤ 0.01 group was −0.006 (and a Spearman’s correlation 0.0003). Based on this observation, the long-range influence detected by our model tended to take place at two codon positions where the ribosome densities were both high. Therefore, there could exist certain associations between two remote codons both with slow elongation rates.

We also discussed possible mechanisms of this long-range influence from remote codons. As shown in S10 Fig, we observed that the long-range contributions tended to be occurred at the codon pairs where donors and acceptors were both with high ribosome densities. In addition, the ribosome densities between donors and acceptors were negatively correlated, with the Pearson’s and Spearman’s correlations of −0.27 and −0.43, respectively. This could be explained by the theory of traffic jams [42]. According to this theory, when a ribosome reaches a slow codon, the elongation rate at this position will be slowed down and the ribosome density will be increased. Meanwhile, fewer ribosomes will continue to traverse to the downstream of the slow codon and the ribosomes at the downstream positions are less likely to pause. We consider two remote codons with high observed ribosome densities, one at the 5′ side while the other at the 3′ side. When traffic jams occurred at the 5′ codon, the ribosome density at the 5′ codon will increase while that at the 3′ codon will decrease. According to another recent study [43], the location with a limited elongation rate results in higher ribosome density on the 5′ side and lower one on the 3′ side. Such an observation can also support our hypothesis. Therefore, the ribosome densities of the two slow and remote codons will be negatively correlated. This hypothesis could well explain our observations in S10 Fig. Our prediction results could thus provide useful biological insights and inspire further experimental investigation.

## Discussion

The translation elongation process is generally regulated by multiple factors and plays an essential role in protein expression. Ribosome profiling techniques provide a great opportunity for measuring elongation dynamics at single codon resolution. In this paper, we developed a deep learning based approach, named RiboMIMO, for modeling the ribosome density distributions of full-length CDS during translation elongation. The RiboMIMO approach accurately predicts the ribosome densities based on the training of ribosome profiling data and outperforms the state-of-the-art baseline methods on both *E.coli* and *S.cerevisiae* datasets. By considering the contextual features of full CDS sequences as well as the relations of ribosome densities between adjacent codons, we also defined an interpretable metric named CIS based on the prediction results of RiboMIMO, to further reveal the relations between different input codons and individual output ribosome densities.

With the help of RiboMIMO, we discovered that the codons located in remote distances from the ribosomal A sites indeed have impact on the prediction of ribosome densities, indicating the potential existence of long-range regulatory mechanisms. In addition, the analyses of CIS values revealed that the ribosome densities can be influenced by several factors, including the positions relative to the ribosomal A sites, codon rarity, wobble base pairing and the adjacent codon pairs, which can be partially supported by the previous studies. The captured contextual patterns by RiboMIMO may provide meaningful hints for understanding the translation elongation mechanisms. Nevertheless, it would be generally hard to directly derive causal conclusions from the model. In fact, translation elongation rates could be affected by many factors associated with codons. In particular, codon usage bias is the major determinant of translation elongation rates [31, 44]. Rare codons usually require more residence time than commonly used codons. Secondary structure of mRNA was also found to regulate translation and folding energy was reported to be negatively correlated with the elongation rates [45–47]. In addition, the GC content or the energy of the folded regions can influence the translation speed [48]. Other factors such as the hydrophobicity and the charge of encoded amino acids could also play important roles in determining the ribosomal velocity [49–51]. Furthermore, the contextual patterns of codons can be recognized during transcription. Thus, transcription may have certain connections with the translation elongation process through the transcription–translation coupling mechanisms [52–56]. Overall, understanding the causalities behind the patterns learned by our model can offer useful insights for further investigating translation dynamics.

The RiboMIMO framework provides a new tool for exploring the regulatory codes of translation elongation through modeling the elongation velocity by a machine learning approach and extracting the contextual determinants of translation efficiency from ribosome profiling data. The CIS derived based on the prediction results of RiboMIMO can accurately evaluate the pair-wise contributions of codons to the predicted ribosome densities, thus providing a detailed portrait of impact maps for arbitrary codon pairs of interest and thus may help reveal the potential causality therein. Nevertheless, there still exist some limitations in the current version of our RiboMIMO framework. First, although our approach benefits from feature embedding of full-CDS sequences and multiple outputs, it is also limited by the sequencing depth of the ribosome profiling data and the number of available genes for training. For a dataset with low sequencing depth, the coverage of each gene would be generally low. In this case, we can only obtain a limited number of genes after filtering in the data preprocessing step. In addition, most of the machine learning based methods, including RiboMIMO, still have limited power in distinguish the artifact in experiments from real biological signals. Although ribosome profiling provided a powerful tool for characterizing the translation elongation dynamics, there may still exist some bias due to the experimental design. For example, ribosome profiling only captures the reads that are protected by monosomes, while ignoring the reads protected by stacked ribosomes (e.g., disomes, trisomes and polysomes). The undetected reads would introduce bias to the distribution of ribosome densities along the coding sequences of mRNAs. This bias could be eliminated in future work through modelling the translation elongation process using polysome profiling information. Nonetheless, RiboMIMO can still provide useful biological insights into understanding the regulatory mechanisms of translation elongation, as demonstrated in our comprehensive tests and analyses.

## Supporting information

S1 Text The performance of RiboMIMO is robust to different evaluation metrics, genomic/sequencing factors, sequence similarities, hyperparameters and cross-dataset evaluation. (PDF)

S1 Fig Scatter plots of averaged RPF counts vs. coverage rates for individual genes for the Mohammad16 (A), Subtelny14 (B), Mohammad19-1 (C) and Mohammad19-2 (D) datasets. (PNG)

S2 Fig Histogram plots of ribosome densities (in log scale) for the Mohammad16 (A), Subtelny14 (B), Mohammad19-1 (C) and Mohammad19-2 (D) datasets. (PNG)

S3 Fig The scatter plots of the predicted vs. the measured ribosome densities for several example genes. (PNG)

S4 Fig Comparisons of different ribosome density prediction methods for the Mohammad16 (A), Subtelny14 (B), Mohammad19-1 (C) and Mohammad19-2 (D) datasets, respectively, in terms of the distributions of the gene-wise Pearson’s correlation coefficients. (PNG)

S5 Fig Contributions of the codon features at different distances to the predicted ribosome densities measured by the codon impact score (CIS) on Mohammad16 (A and C) and Mohammad19-2 (B and D) datasets. (PNG)

S6 Fig Correlations of the ribosome densities and the codon impact scores derived from RiboMIMO at the A sites (A–B) and E sites (C–D) with codon adaptation index on the Mohammad16 dataset in *E.coli*. (PNG)

S7 Fig Correlations of the ribosome densities and the codon impact scores derived from RiboMIMO with other biological factors including codon adaptation index, tRNA adaptation index, GC content, mRNA folding energy and amino acid hydrophobicity on the Mohammad19-1 (*E.coli*) and Subtelny14 (*S.cerevisiae*) datasets. (PNG)

S8 Fig The codon-distance impact score (CIS_*c*,*d*_) (A–B) and amino acid-distance impact score (CIS_aa,*d*_) (C–F) on Mohammad16 (A and C), Subtelny14 (D), Mohammad19-1 (E) and Mohammad19-2 (B and F) datasets, respectively. (PNG)

S9 FigThe long-range codon impact score (${\text{CIS}}_{c_{1},c_{2}}^{\left(\text{remote}\right)}$) on the Mohammad16, Mohammad19-1 and Mohammad19-2 datasets.(PNG)

S10 Fig Distributions of the predicted ribosome densities of long-range donor-acceptor pairs on the Subtelny14 dataset. (PNG)

S1 Table Statistics of the ribosome profiling data used in this study. (XLSX)

S2 Table Architecture details and hyperparameters of the RiboMIMO framework. (XLSX)

S3 Table The list of 17 adjacent codon pairs identified by [39] to have inhibitory effects on the translation efficiency in yeast. The direction is from 5′ to 3′. (XLSX)
