# Supplementary material for: Full-length ribosome density prediction by a multi-input and multi-output model
Source: PLoS Comput Biol. 2021 Mar 26;17(3):e1008842. doi: 10.1371/journal.pcbi.1008842 (PMC8026034; doi:10.1371/journal.pcbi.1008842)
Supplement: S1 Text — (PDF) [file pcbi.1008842.s001.pdf]

S1 Text for
Full-length ribosome density prediction by a multi-input multi-output model

A The performance of RiboMIMO is robust to evaluation metrics.

In addition to gene-wise Pearson's correlations (i.e., one correlation for one gene) used in the main text, other evaluation metrics were also used to assess the performance of our model. We first used Pearson's correlations across 10-fold cross-validation (i.e., one correlation for one fold) to examine the variation of prediction results. As shown in Table A, the standard deviations were computed across 10 folds and can reflect the variability of correlations across 10-fold cross-validation. The conclusions here were consistent with Table 1.

In the main text, we used Fridman test and Dunn's multiple comparison tests as non-parametric statistical tests to compare the performances of different methods or hyperparameter settings. Fridman test and Dunn's multiple comparison tests use ranks to evaluate the significant levels and estimate the corresponding p-values. We also used the one-way ANOVA (analysis of variance) with the Geisser-Greenhouse correction as well as Tucky's multiple comparison tests to evaluate the difference of prediction results. We reported the mean and the corresponding 95% confidence interval (CI) as the estimator and the uncertainty measure, respectively. As shown in Table B, the conclusions using one-way ANOVA were consistent with those using Fridman tests (Table 1). In addition, the 95% CI can also provide an appropriate measure of uncertainty and seemed consistent with the results of statistical tests.

Spearman's correlation could also provide a good metric in addition to the Pearson's correlation. We also calculated the Spearman's correlation for each method. As shown in Table C, the results using Spearman's correlations were consistent with those using Pearson's correlations (Table 1). The performance of our RiboMIMO significantly outperformed the baseline methods in terms of either Pearson's or Spearman's correlation coefficients. Therefore, both correlation scores (i.e., Pearson's and Spearman's correlations) can serve as reliable evaluation metrics for

comparing the performances of different models.

B Relation between the performance of RiboMIMO and genomic/sequencing factors.

To take a closer look on the performances of our RiboMIMO model, we compared the gene-wise correlations obtained for individual genes in the test set at each fold of cross-validation, with several genomic/sequencing factors including GC content, CDS length, averaged RPF counts and coverage rate (Fig A). The averaged RPF counts can be used to measure the gene expression levels. The coverage rate indicates the quality of the ribo-seq data, as zero counts are regarded as missing data instead of real zeros. We observed that, the gene expression levels (measured by averaged RPF counts) were correlated with GC content, CDS lengths and coverage rates (already shown in S1 Fig). Within all the genes in the dataset, those genes with higher coverage rate and higher averaged RPF counts were more likely to be predicted accurately, regardless of their overall GC content or CDS lengths. The highly expressed genes are more likely to be detected by the ribosome profiling technique, with high coverage rates and thus better quality. Under this circumstance, it was natural that the genes with higher coverage rates or averaged RPF counts were associated with better prediction accuracy. Noted that although the GC content and CDS lengths were correlated with the averaged RPF counts, the performance of our model was not influenced by these factors. In summary, the performance of our model was robust to CDS lengths and GC content, and only influenced by the quality of ribo-seq data measured by coverage rates.

C The performance of RiboMIMO is robust to sequence similarities.

Noted that there may exist a risk from which the potential correlations between genes in training and test data may result in a misleading good accuracy of our method. Here, we proved that this was not the case. We first calculated the Smith-Waterman similarity between the sequences of each two genes in the dataset using a sequence alignment software [1]. Then we split the whole dataset into 10 folds by ensuring that the maximum similarities between any two genes in different folds were below a threshold. We retrained our RiboMIMO model using different thresholds (i.e., 0.2, 0.3, 0.4 and 0.5) to see whether the similarities between genes from the

training and test sets could influence the performance (Table D). In Table D, we observed no significant difference between the results by randomly splitting the train/test data and splitting according to the maximum similarities. Therefore, the performance of our RiboMIMO model was not influenced by the train/test splitting scheme.

D The performance of RiboMIMO is robust to the selection of hyperparameters in the dual-task module.

There are two hyperparameters in the dual-task module, that is, discretization thresholds of classification labels and the weight of the classification loss relative to the regression loss (i.e., α). We first examined the performance of RiboMIMO with respect to different threshold values as well as the numbers of thresholds. As shown in Table E, the threshold 0 (i.e., μ) performed the best among single thresholds. The thresholds of 0,2 (μ, $\mu + 2\sigma$) with three classes performed better than single thresholds. The differences of the thresholds with two or more classes compared with threshold 0 (μ) were all not significant. Therefore, our original selection (μ, $\mu + 2\sigma$) can be considered a good setting and the model was robust to different choices of the thresholds. We also showed the performance with different values of α (Table F). We observed that our model was robust to different α values and $\alpha = 1$ yielded good performance for all datasets.

E Generalization of RiboMIMO on replicated and independent datasets.

To show the performance of RiboMIMO trained on one dataset and tested on another, we conducted a cross-dataset evaluation analysis. For a fair comparison, we only selected the overlapped genes among the three datasets (i.e., Mohammad16, Mohammad19-1, Mohammad19-2) of *E.coli* for training and testing, resulting in 792 genes. The evaluation was conducted using a 10-fold cross-validation procedure. We observed that the performance of our RiboMIMO model trained on one dataset and tested on another dataset was approximately upper bounded by the correlation between the two datasets (Table G). Noted that the two datasets Mohammad19-1 and Mohammad19-2 were biological replicates and had the highest correlation (also called data correlation) among all pairs of the datasets. The cross-dataset performance on these two datasets was quite close to the data correlation, thus indicating the good generalization ability of our model. For the Mohammad16 and Mohammad19 datasets, the dataset correlations were relatively low.

Therefore, the cross-dataset evaluation cannot provide meaningful results due to the intrinsic inconsistence in experimental data. These results may indicate that the translation elongation dynamics itself is not well correlated among different conditions or protocols, and thus it will not be feasible to use a single model to represent the features in all conditions. Nevertheless, our model can still perform good prediction with high accuracy under similar conditions, indicating its good generalization ability to predict the ribosome densities.

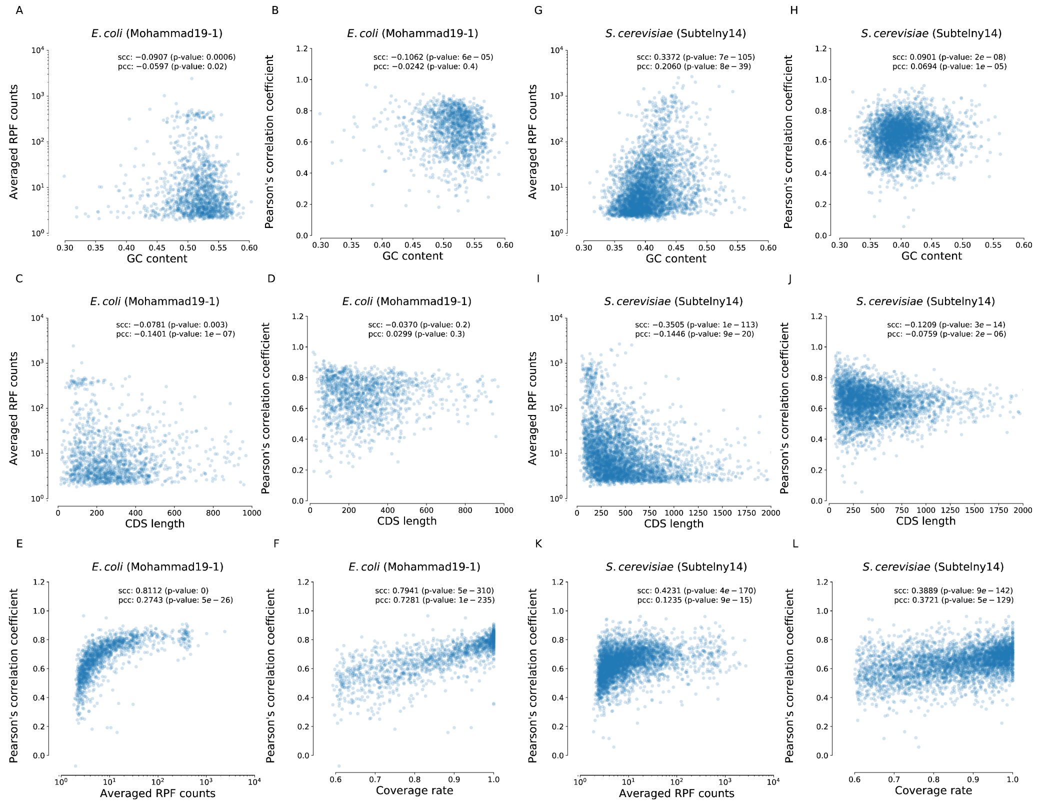

Fig A. Scatter plots of gene-wise correlations with biophysical factors including GC content, CDS lengths, averaged RPF counts and coverage rates on the Mohammad19-1 dataset of *E.coli* (A–F) and the Subtelny14 dataset of *S.cerevisiae* (G–L).

Table A. Performance evaluation of different prediction methods, measured in terms of the Pearson's correlation coefficient across 10-fold cross-validation.

**Methods**	**Dataset**			
	**Mohammad16**	**Subtelny14**	**Mohammad19-1**	**Mohammad19-2**
riboShape	0.3301 ± 0.0145 (****)	0.2956 ± 0.0064 (****)	0.2536 ± 0.0135 (****)	0.2288 ± 0.0135 (****)
RUST	0.5319 ± 0.0066 (****)	0.3807 ± 0.0038 (****)	0.5480 ± 0.0099 (****)	0.5585 ± 0.0187 (****)
iXnos	0.6144 ± 0.0050 (****)	0.5406 ± 0.0076 (****)	0.6307 ± 0.0083 (****)	0.6548 ± 0.0181 (****)
RiboMIMO	**0.6817 ± 0.0125**	**0.6304 ± 0.0109**	**0.6822 ± 0.0102**	**0.6959 ± 0.0224**
w/o nt & aa	0.6656 ± 0.0075 (***)	0.6251 ± 0.0099 (**)	0.6781 ± 0.0112 (ns)	0.6910 ± 0.0216 (**)
w/o nt	0.6768 ± 0.0121 (ns)	0.6286 ± 0.0100 (ns)	0.6792 ± 0.0101 (*)	0.6918 ± 0.0198 (ns)
w/o aa	0.6743 ± 0.0105 (*)	0.6263 ± 0.0101 (ns)	0.6810 ± 0.0119 (ns)	0.6955 ± 0.0208 (ns)
w/o dual-task	0.6672 ± 0.0085 (***)	0.6191 ± 0.0085 (***)	0.6751 ± 0.0095 (***)	0.6842 ± 0.0227 (****)

10-fold cross-validation through randomly splitting genes into training and test sets was performed on the four datasets to assess the performance of each method. The gene-wise correlations were computed by comparing the predicted and measured ribosome densities for individual genes in the test set at each 10-fold cross-validation. The mean ± SD of the Pearson's correlation coefficients across 10-folds are shown. Significant levels from one-way ANOVA with Dunnett's multiple comparison correction are shown in the parentheses. The statistical tests were conducted by comparing different baseline methods or the RiboMIMO model without certain modules with the original RiboMIMO model. ****: $P < 0.0001$, ***: $P < 0.001$, **: $P < 0.01$, *: $P < 0.05$, ns: not significant. nt and aa stand for nucleotide encoding and amino acid encoding, respectively. The best results are shown in bold.

Table B. Performance evaluation of different prediction methods, measured in terms of the Pearson's correlation coefficient.

Methods	Dataset	
	**Mohammad16**	**Subtelny14**
riboShape	0.4308 [0.4244, 0.4372] (****)	0.3892 [0.3861, 0.3923] (****)
RUST	0.5700 [0.5626, 0.5773] (****)	0.4151 [0.4119, 0.4184] (****)
iXnos	0.6325 [0.6257, 0.6382] (****)	0.5625 [0.5593, 0.5657] (****)
RiboMIMO	**0.6904 [0.6876, 0.7001]**	**0.6385 [0.6353, 0.6416]**
w/o nt & aa	0.6750 [0.6685, 0.6815] (****)	0.6312 [0.6280, 0.6344] (****)
w/o nt	0.6892 [0.6828, 0.6957] (*)	0.6351 [0.6320, 0.6383] (****)
w/o aa	0.6867 [0.6802, 0.6933] (****)	0.6345 [0.6313, 0.6376] (****)
w/o dual-task	0.6791 [0.6726, 0.6856] (****)	0.6263 [0.6232, 0.6295] (****)
**Methods**	**Dataset**	
	**Mohammad19-1**	**Mohammad19-2**
riboShape	0.3312 [0.3245, 0.3380] (****)	0.2879 [0.2822, 0.2936] (****)
RUST	0.5622 [0.5552, 0.5692] (****)	0.5775 [0.5711, 0.5839] (****)
iXnos	0.6333 [0.6267, 0.6400] (****)	0.6573 [0.6511, 0.6636] (****)
RiboMIMO	**0.6706 [0.6630, 0.6772]**	**0.6937 [0.6871, 0.7003]**
w/o nt & aa	0.6674 [0.6604, 0.6744] (**)	0.6885 [0.6818, 0.6951] (****)
w/o nt	0.6683 [0.6615, 0.6752] (ns)	0.6897 [0.6830, 0.6963] (****)
w/o aa	0.6706 [0.6637, 0.6776] (ns)	0.6935 [0.6868, 0.7001] (ns)
w/o dual-task	0.6651 [0.6581, 0.6721] (****)	0.6833 [0.6767, 0.6899] (****)

10-fold cross-validation through randomly splitting genes into training and test sets was performed on the four datasets to assess the performance of each method. The gene-wise correlations were computed by comparing the predicted and measured ribosome densities for individual genes in the test set at each 10-fold cross-validation. The mean and the corresponding 95% confidence interval (CI) of the gene-wise Pearson's correlation coefficients across the whole dataset are shown. The values of lower 95% CI and upper 95% CI are shown in the square brackets. Significant levels from one-way ANOVA with Tucky's multiple comparison correction are shown in the parentheses. The statistical tests were conducted by comparing different baseline methods or the RiboMIMO model without certain modules with the original RiboMIMO model. ****: $P < 0.0001$, ***: $P < 0.001$, **: $P < 0.01$, *: $P < 0.05$,

ns: not significant. nt and aa stand for nucleotide encoding and amino acid encoding, respectively. The best results are shown in bold.

Table C. Performance evaluation of different prediction methods, measured in terms of the Spearman's correlation coefficient.

**Methods**	**Dataset**			
	**Mohammad16**	**Subtelny14**	**Mohammad19-1**	**Mohammad19-2**
riboShape	0.4549 ± 0.1072 (****)	0.3896 ± 0.0977 (****)	0.3354 ± 0.1219 (****)	0.2814 ± 0.1098 (****)
RUST	0.6274 ± 0.0876 (****)	0.4360 ± 0.0955 (****)	0.5852 ± 0.1393 (****)	0.5997 ± 0.1304 (****)
iXnos	0.6532 ± 0.0890 (****)	0.5243 ± 0.0940 (****)	0.6320 ± 0.1367 (****)	0.6540 ± 0.1301 (****)
RiboMIMO	**0.7186 ± 0.0825**	**0.5879 ± 0.0953**	**0.6641 ± 0.1434**	**0.6872 ± 0.1335**
w/o nt & aa	0.6982 ± 0.0819 (****)	0.5834 ± 0.0961 (****)	0.6622 ± 0.1407 (****)	0.6839 ± 0.1335 (****)
w/o nt	0.7162 ± 0.0817 (ns)	0.5854 ± 0.0956 (****)	0.6629 ± 0.1399 (**)	0.6840 ± 0.1332 (****)
w/o aa	0.7100 ± 0.0830 (****)	0.5873 ± 0.0969 (ns)	0.6650 ± 0.1397 (ns)	0.6872 ± 0.1333 (ns)
w/o dual-task	0.6999 ± 0.0832 (****)	0.5743 ± 0.0942 (****)	0.6588 ± 0.1417 (****)	0.6771 ± 0.1327 (****)

10-fold cross-validation through randomly splitting genes into training and test sets was performed on the four datasets to assess the performance of each method. The gene-wise correlations were computed by comparing the predicted and measured ribosome densities for individual genes in the test set at each 10-fold cross-validation. The mean ± SD of the gene-wise Spearman's correlation coefficients across the whole dataset are shown. Significant levels from Friedman tests with Dunn's multiple comparison correction are shown in the parentheses. The statistical tests were conducted by comparing different baseline methods or the RiboMIMO model without certain modules with the original RiboMIMO model. ****: $P < 0.0001$, ***: $P < 0.001$, **: $P < 0.01$, *: $P < 0.05$, ns: not significant. nt and aa stand for nucleotide encoding and amino acid encoding, respectively. The best results are shown in bold.

Table D. Performance evaluation of RiboMIMO using different train/test dataset splitting schemes, measured in terms of the Pearson's correlation coefficient.

Dataset splitting scheme	Dataset			
	**Mohammad16**	**Subtelny14**	**Mohammad19-1**	**Mohammad19-2**
Random	0.6938 ± 0.1100	0.6385 ± 0.0997	0.6706 ± 0.1353	0.6937 ± 0.1345
Threshold 0.2	0.6933 ± 0.1130 (ns)	0.6368 ± 0.1016 (ns)	0.6713 ± 0.1349 (ns)	0.6953 ± 0.1352 (ns)
Threshold 0.3	0.6913 ± 0.1108 (ns)	0.6368 ± 0.0997 (ns)	0.6711 ± 0.1348 (ns)	0.6937 ± 0.1348 (ns)
Threshold 0.4	0.6909 ± 0.1122 (ns)	0.6365 ± 0.0997 (ns)	0.6717 ± 0.1352 (ns)	0.6933 ± 0.1352 (ns)
Threshold 0.5	0.6908 ± 0.1102 (ns)	0.6367 ± 0.0996 (ns)	0.6715 ± 0.1354 (ns)	0.6932 ± 0.1346 (ns)

10-fold cross-validation through randomly splitting genes into training and test sets was performed on the four datasets to assess the performance of each threshold. The gene-wise correlations were computed by comparing the predicted and measured ribosome densities for individual genes in the test set at each 10-fold cross-validation. The mean ± SD of the gene-wise Pearson's correlation coefficients across the whole dataset are shown. Significant levels from the Friedman tests with Dunn's multiple comparison correction are also shown in the parentheses. The statistical tests were conducted by comparing the RiboMIMO model with randomly split train/test data to those using different similarity thresholds for splitting the data. ****: $P < 0.0001$, ***: $P < 0.001$, **: $P < 0.01$, *: $P < 0.05$, ns: not significant.

Table E. Performance evaluation of RiboMIMO using different discretization thresholds, measured in terms of the Pearson's correlation coefficient.

Threshold	Dataset			
	**Mohammad16**	**Subtelny14**	**Mohammad19-1**	**Mohammad19-2**
-3	0.6831 ± 0.1122 (****)	0.6288 ± 0.1025 (****)	0.6696 ± 0.1330 (ns)	0.6909 ± 0.1343 (****)
-2	0.6838 ± 0.1121 (****)	0.6285 ± 0.1023 (****)	0.6686 ± 0.1347 (**)	0.6909 ± 0.1336 (****)
-1	0.6899 ± 0.1126 (ns)	0.6305 ± 0.1015 (****)	0.6689 ± 0.1329 (*)	0.6892 ± 0.1351 (****)
0	0.6905 ± 0.1105 (ns)	0.6360 ± 0.0992 (**)	0.6688 ± 0.1355 (ns)	0.6908 ± 0.1348 (****)
1	0.6886 ± 0.1116 (**)	0.6343 ± 0.1018 (****)	0.6684 ± 0.1345 (*)	0.6899 ± 0.1346 (****)
2	0.6838 ± 0.1116 (****)	0.6283 ± 0.1007 (****)	0.6659 ± 0.1354 (****)	0.6884 ± 0.1344 (****)
3	0.6795 ± 0.1138 (****)	0.6265 ± 0.1043 (****)	0.6683 ± 0.1343 (**)	0.6885 ± 0.1337 (****)
0,2	0.6938 ± 0.1100	0.6385 ± 0.0997	0.6706 ± 0.1353	0.6937 ± 0.1345
0,3	0.6940 ± 0.1093 (ns)	0.6387 ± 0.1005 (ns)	0.6715 ± 0.1350 (ns)	0.6947 ± 0.1346 (ns)
0,2,-2	0.6947 ± 0.1107 (ns)	0.6378 ± 0.0993 (ns)	0.6726 ± 0.1340 (ns)	0.6953 ± 0.1351 (**)
0,3,-3	0.6915 ± 0.1099 (ns)	0.6375 ± 0.1000 (ns)	0.6721 ± 0.1368 (ns)	0.6962 ± 0.1343 (****)

10-fold cross-validation through randomly splitting genes into training and test sets was performed on the four datasets to assess the performance of the model with each threshold. The gene-wise correlations were computed by comparing the predicted and measured ribosome densities for individual genes in the test set at each 10-fold cross-validation. The mean ± SD of the gene-wise Pearson's correlation coefficients across the whole dataset are shown. Significant levels from the Friedman tests with Dunn's multiple comparison correction are also shown in the parentheses. The statistical tests were conducted by comparing the results with individual thresholds with that with threshold 0,2. The number in the "Threshold" column indicates the number of standard deviations relative to the mean used for discretization. For example, "0,2" stands for the thresholds of $(\mu,\ \mu + 2\sigma)$ with three classes. ****: $P < 0.0001$, ***: $P < 0.001$, **: $P < 0.01$, *: $P < 0.05$, ns: not significant.

Table F. Performance evaluation of RiboMIMO using different weights of the classification loss, measured in terms of the Pearson's correlation coefficient.

α	**Dataset**			
	**Mohammad16**	**Subtelny14**	**Mohammad19-1**	**Mohammad19-2**
0.1	0.6811 ± 0.1148 (****)	0.6298 ± 0.1021 (****)	0.6658 ± 0.1356 (****)	0.6873 ± 0.1355 (****)
0.3	0.6824 ± 0.1124 (****)	0.6319 ± 0.1009 (****)	0.6686 ± 0.1327 (**)	0.6908 ± 0.1356 (****)
1	0.6938 ± 0.1100	0.6385 ± 0.0997	0.6706 ± 0.1353	0.6937 ± 0.1345
3	0.6992 ± 0.1082 (ns)	0.6389 ± 0.1013 (ns)	0.6724 ± 0.1366 (ns)	0.6945 ± 0.1348 (ns)
10	0.7135 ± 0.1044 (****)	0.6362 ± 0.0990 (**)	0.6683 ± 0.1375 (*)	0.6921 ± 0.1353 (**)

10-fold cross-validation through randomly splitting genes into training and test sets was performed on the four datasets to assess the performance of the model with each α setting. The gene-wise correlations were computed by comparing the predicted and measured ribosome densities for individual genes in the test set at each 10-fold cross-validation. The mean ± SD of the gene-wise Pearson's correlation coefficients across the whole dataset are shown. Significant levels from the Friedman tests with Dunn's multiple comparison correction are also shown in the parentheses. The statistical tests were conducted by comparing the RiboMIMO model with $\alpha = 1$ with those with other α values. ****: $P < 0.0001$, ***: $P < 0.001$, **: $P < 0.01$, *: $P < 0.05$, ns: not significant. RiboMIMO was robust to different α values and $\alpha = 1$ yielded good performance for all datasets.

Table G. Cross-dataset performance evaluation of RiboMIMO, measured in terms of the Pearson's correlation coefficient.

Dataset for training	Dataset for testing	Gene-wise correlation	Data correlation
Mohammad16	Mohammad16	0.8064 ± 0.1080	
	Mohammad19-1	0.0218 ± 0.0918	0.0242
	Mohammad19-2	0.0445 ± 0.0928	0.0389
Mohammad19-1	Mohammad16	0.0187 ± 0.0914	0.0242
	Mohammad19-1	0.7379 ± 0.1059	
	Mohammad19-2	0.5987 ± 0.1073	0.5926
Mohammad19-2	Mohammad16	0.0345 ± 0.0940	0.0389
	Mohammad19-1	0.5657 ± 0.1161	0.5926
	Mohammad19-2	0.7653 ± 0.0979	

10-fold cross-validation was performed on each dataset for performance evaluation. The mean and standard deviation (mean ± SD) of the gene-wise correlations across all the overlapped genes are shown.

References

[1] Zhao M, Lee WP, Garrison EP, Marth GT. SSW library: an SIMD Smith-Waterman C/C++ library for use in genomic applications. PloS one. 2013;8(12).
