# Supplementary figures and images for: Full-length ribosome density prediction by a multi-input and multi-output model
Source: PLoS Comput Biol. 2021 Mar 26;17(3):e1008842. doi: 10.1371/journal.pcbi.1008842 (PMC8026034; doi:10.1371/journal.pcbi.1008842)

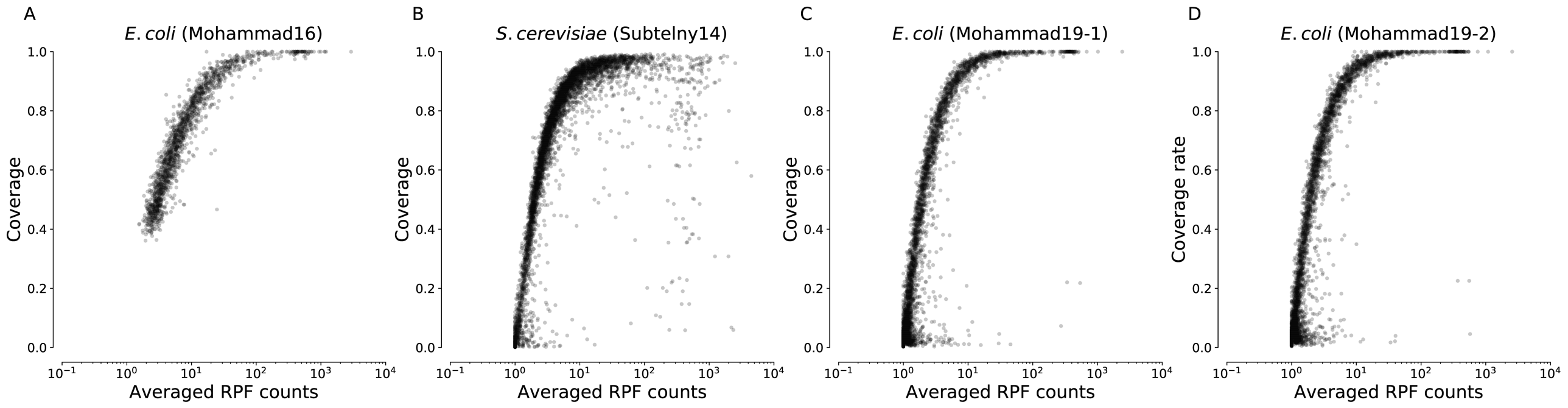

Supplement: S1 Fig — (PNG) [file pcbi.1008842.s002.png]

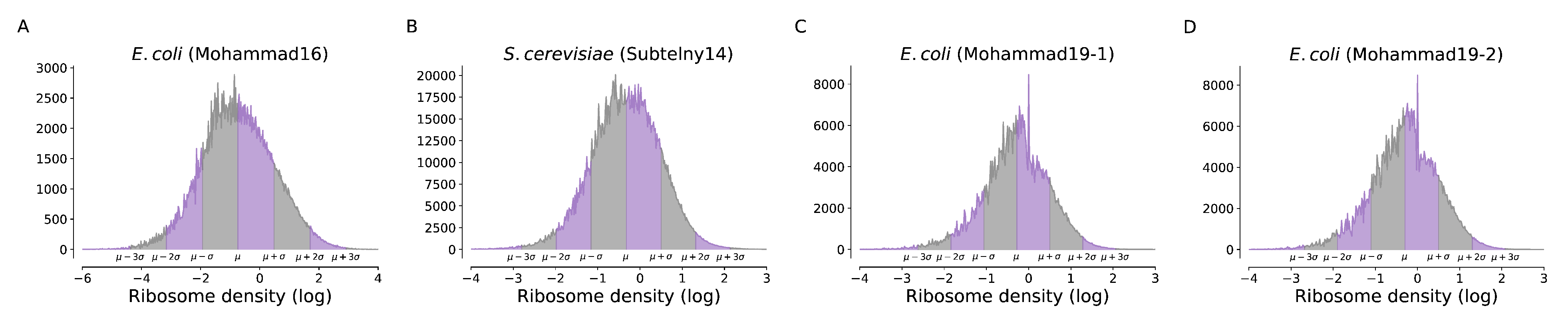

Supplement: S2 Fig — (PNG) [file pcbi.1008842.s003.png]

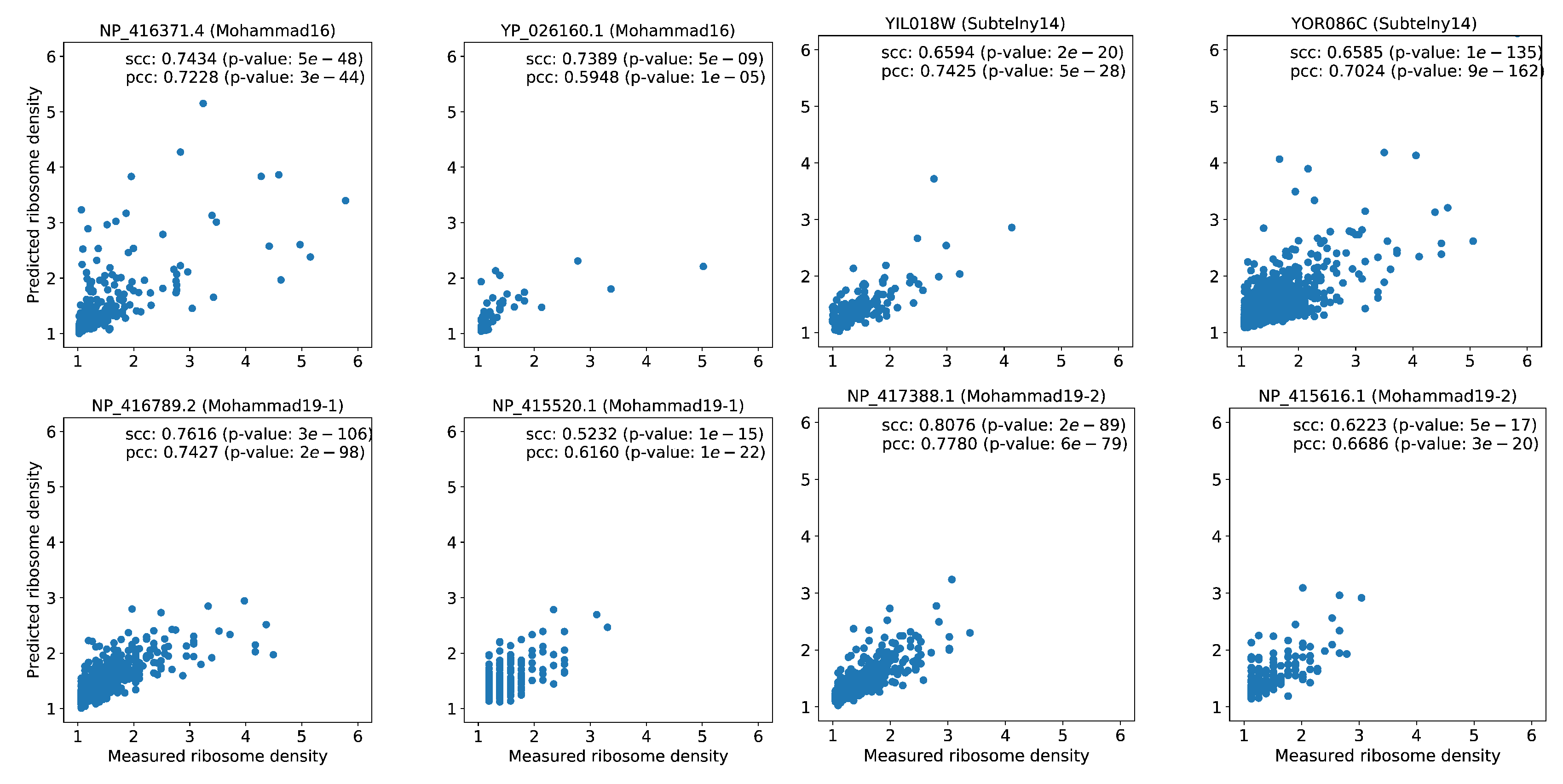

Supplement: S3 Fig — (PNG) [file pcbi.1008842.s004.png]

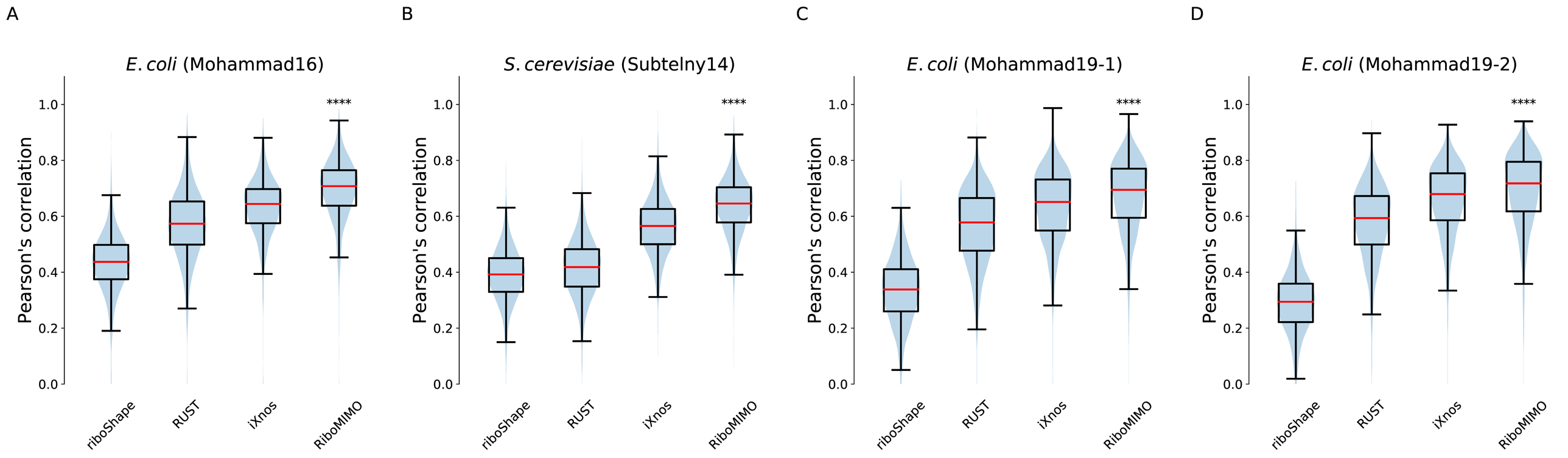

Supplement: S4 Fig — (PNG) [file pcbi.1008842.s005.png]

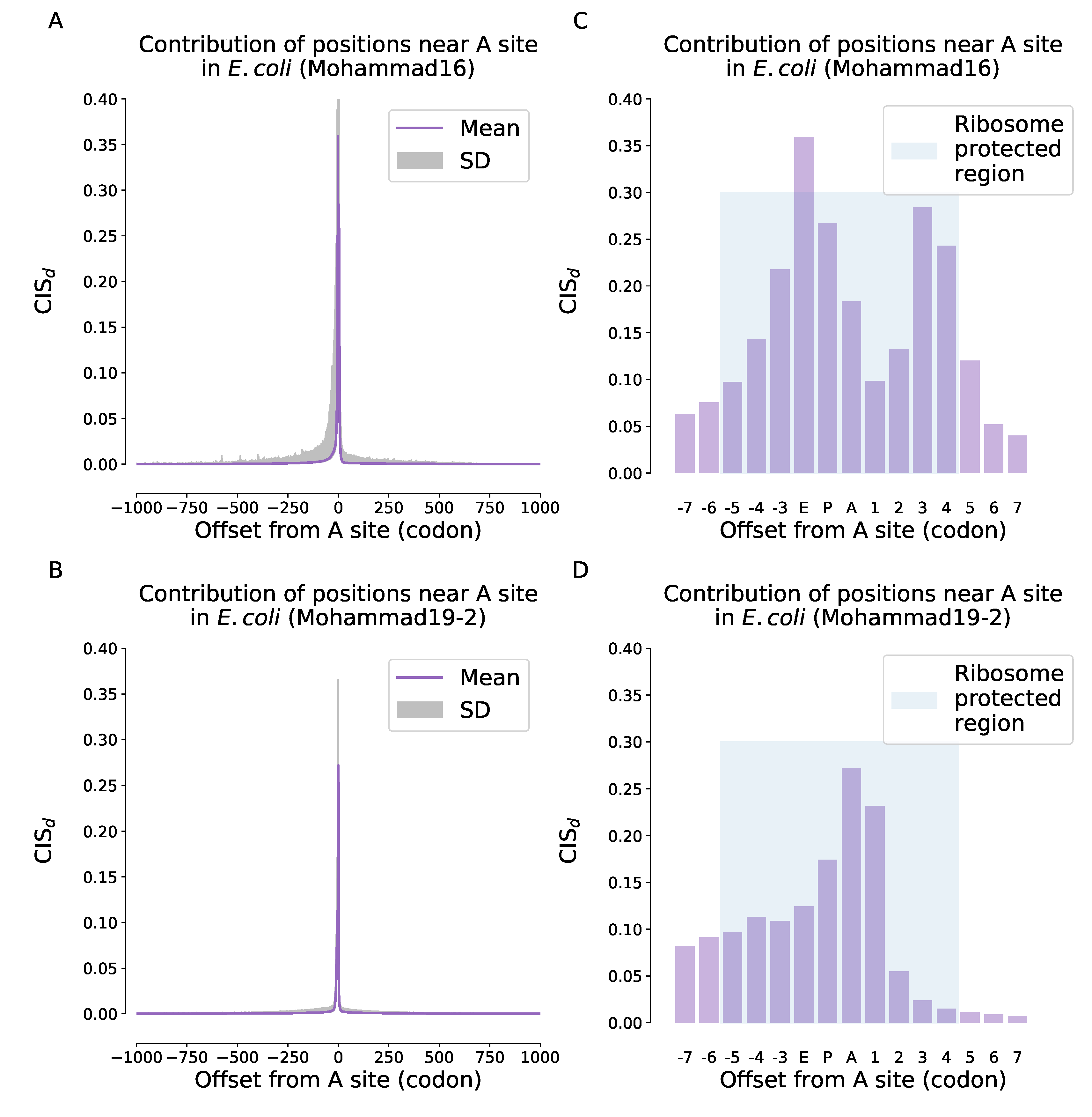

Supplement: S5 Fig — (PNG) [file pcbi.1008842.s006.png]

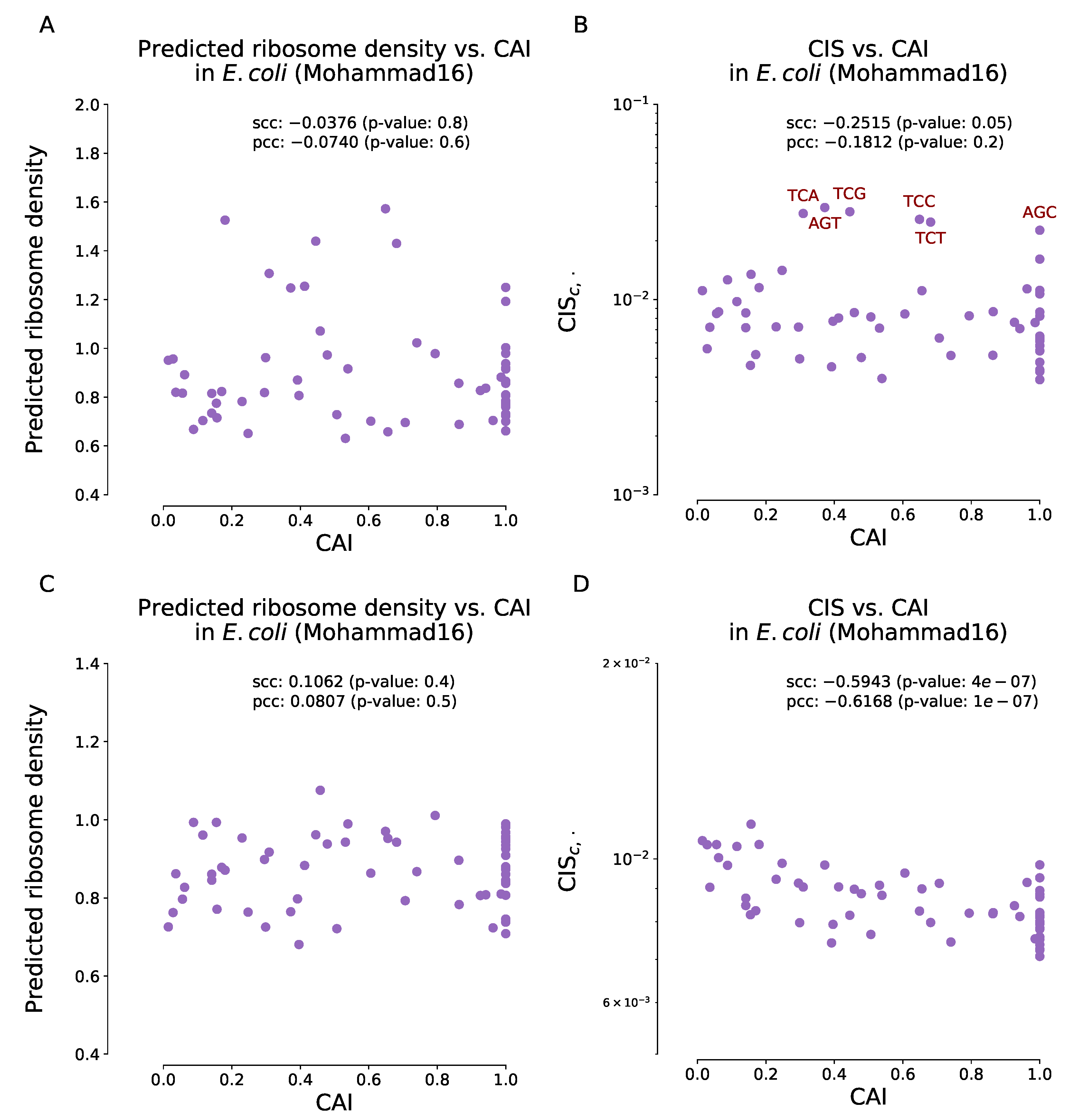

Supplement: S6 Fig — (PNG) [file pcbi.1008842.s007.png]

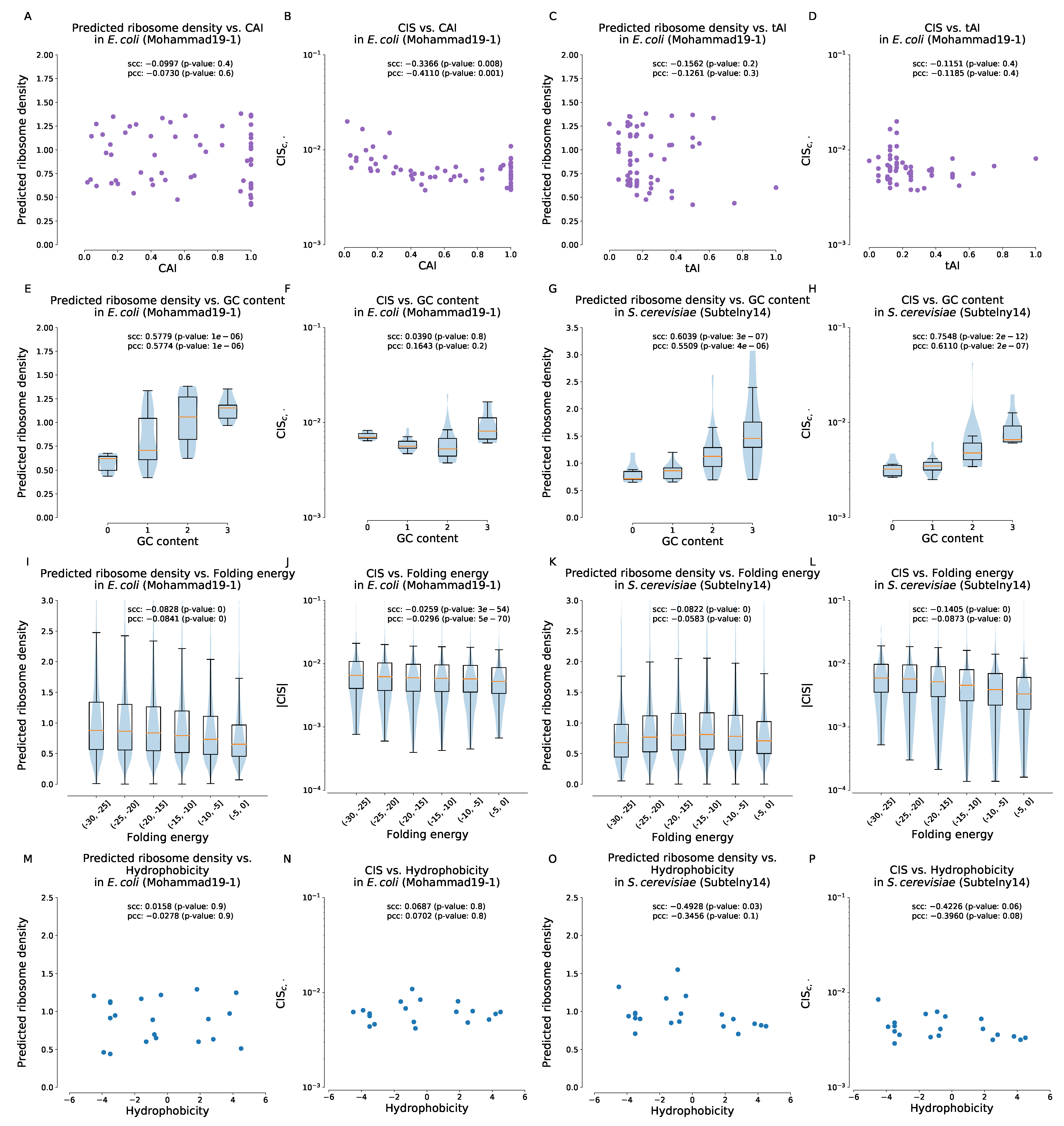

Supplement: S7 Fig — (PNG) [file pcbi.1008842.s008.png]

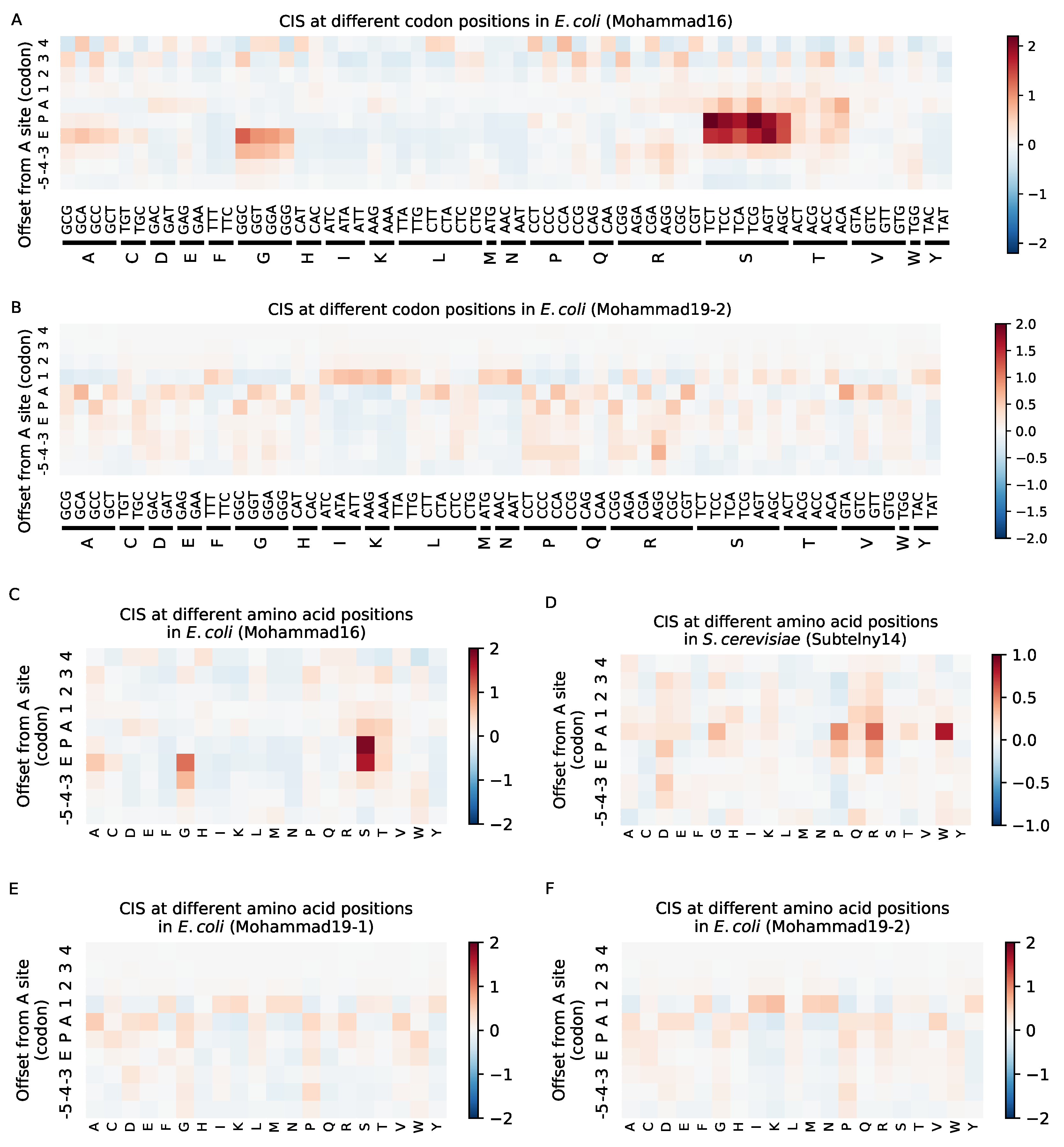

Supplement: S8 Fig — (PNG) [file pcbi.1008842.s009.png]

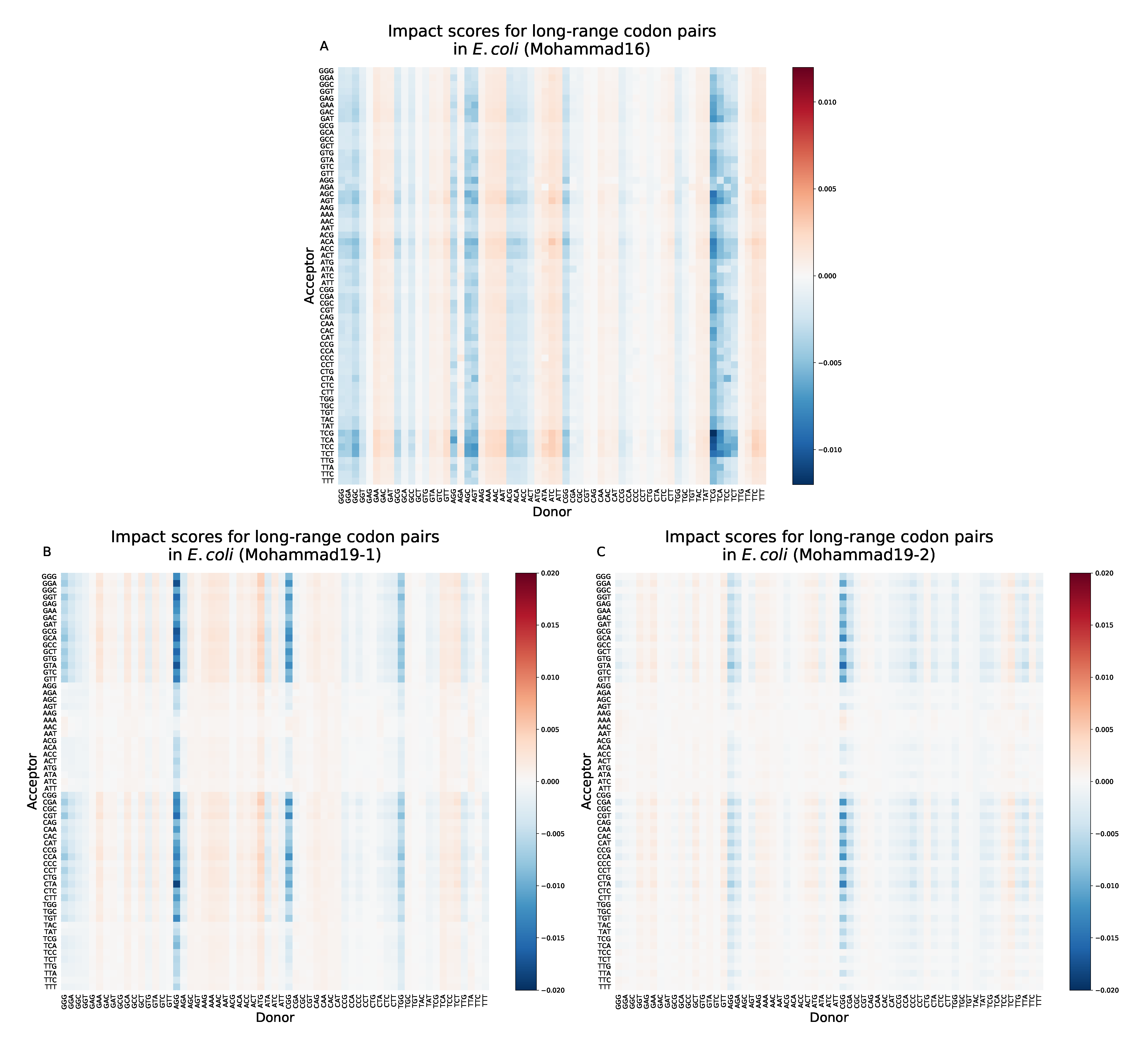

Supplement: S9 Fig — (PNG) [file pcbi.1008842.s010.png]

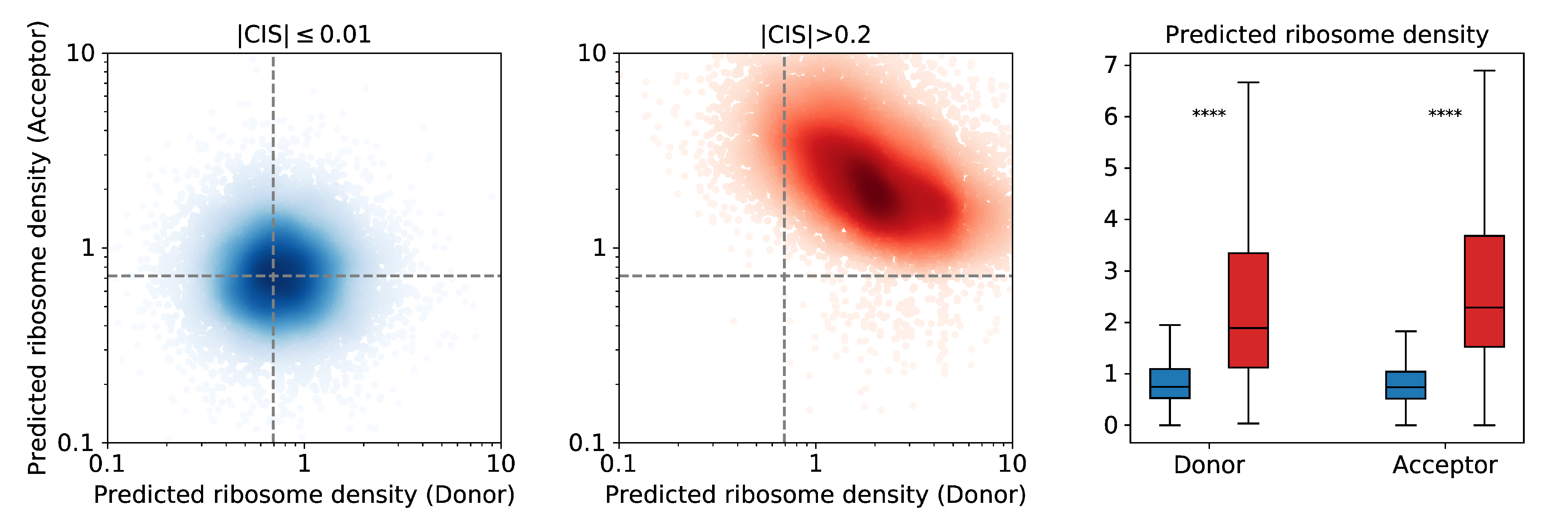

Supplement: S10 Fig — (PNG) [file pcbi.1008842.s011.png]
